# Chemical-mediated alteration of antibiotic susceptibility: mechanisms and potential new targets for antibiotic adjuvant discovery

**DOI:** 10.1128/jb.00351-25

**Published:** 2026-01-12

**Authors:** Joshua M. E. Adams, Omar M. El-Halfawy

**Affiliations:** 1 Department of Chemistry and Biochemistry, Faculty of Science, University of Regina 98641, Regina, Saskatchewan, Canada; Geisel School of Medicine at Dartmouth, Hanover, New Hampshire, USA

**Keywords:** volatile organic compounds, indole, polyamines, antibiotic adjuvants, antimicrobial resistance, chemical-mediated resistance, phenylacetic acid, *Pseudomonas* quinolone signal

## Abstract

The critical rise in antimicrobial resistance rates, coupled with a nearly stagnant drug discovery pipeline, has resulted in a sharp surge in clinical failure of antibiotic therapy. Antimicrobial resistance, possibly leading to therapeutic failures, has been predominantly attributed to genetically encoded intrinsic or acquired resistance determinants. However, small molecules that bacteria may be exposed to at the infection site have been implicated in antibiotic resistance, tolerance, and persister formation. Such chemicals, either produced by the host or bacteria, typically elicit transient effects on antibiotic responses while bacteria are exposed to them; as such, their effects may alter therapeutic outcomes but would not be detected in standard *in vitro* antibiotic susceptibility tests. Chemicals produced by certain bacteria may alter the response of the same or other bacterial species at the site of infection, potentially providing communal protection from antibiotic treatment. Notably, biosynthesis, uptake, and response mechanisms to such chemicals are genetically encoded; thus, these determinants may constitute potential drug targets to circumvent chemical-mediated resistance. This review aims to provide an update on small molecules that alter antibiotic responses, the molecular mechanisms thereof, and the recent progress made in the discovery of antibiotic adjuvants targeting these pathways.

## INTRODUCTION

The overuse and misuse of antibiotics in the interconnected One Health human, animal, and environmental triad have accelerated the emergence of antimicrobial resistance (AMR) (1). This critical rise in AMR, alongside the concurrent slow and expensive drug discovery pipeline, has resulted in a sharp rise in clinical failure rates of antibiotic therapy (2, 3). If the current trends continue, AMR has been projected to cause an attributable cumulative death toll of ~40 million deaths worldwide between the years 2025 and 2050 (4).

Antibiotic therapeutic failures are often ascribed to conventional resistance mechanisms, yet emerging evidence indicates that small molecules present at the infection site can influence antibiotic resistance, tolerance, and persister formation. *Bona fide* resistance determinants such as specific antibiotic-modifying enzymes, efflux pumps, and impermeable cell membranes lead to stable, high levels of antibiotic resistance (5, 6). Efforts to combat AMR have predominantly focused on defining and targeting such resistance mechanisms (6, 7). However, a dynamic network of signaling compounds and other small molecules—secreted by bacteria or produced by mammalian host cells—has been shown to influence bacterial responses to antibiotics (8). These chemical-mediated effects often lead to transient AMR occurring while bacteria are exposed to such small molecules, which may still alter the antibiotic therapeutic outcomes, potentially underlying some of the antibiotic therapeutic failures. Notably, the effects of many of the chemicals likely present at infection sites on bacterial responses to antibiotics are typically not captured under the conditions of standard antimicrobial susceptibility testing (AST) assays, which may explain, in part, some instances of discordance between AST results and antibiotic therapeutic outcomes (9).

Targeting biosynthesis, uptake, and response mechanisms to such chemicals expands the potential list of druggable targets; inhibitors of such targets may serve as antibiotic adjuvants that improve therapeutic outcomes of current antibiotic treatments. These potential targets are typically non-essential and are involved in antibiotic resistance as a result of the chemical landscape at the infection site. Given that inhibitors thereof would suppress bacterial growth only when combined with antibiotics, resistance to these drug combinations could still be derived from selective pressure at the site of infection. Resistance to various antibiotic adjuvants has been detected; for example, resistance to various β-lactam antibiotic adjuvants, including β-lactamase inhibitors (reviewed in reference 10) and an inhibitor of the envelope stress response regulator GraR (11), was more readily selected for upon exposure of bacteria to the antibiotic-antibiotic adjuvant combinations (10, 11). Yet, we posit that inhibitors acting as antibiotic adjuvants may have reduced selective pressure to drive resistance when present alone outside of the infection site in environmental settings. This feature may help mitigate the acquisition of resistance due to environmental exposure, which is a major contributor to the continual development of AMR (12).

This review will shed light on chemical-mediated alteration of antimicrobial susceptibility and the molecular basis thereof, covering the significant advancements made in this area since we reviewed it in 2012 (8). Chemicals covered include polyamines, *Pseudomonas* quinolone signal (PQS), phenylacetic acid (PAA), indole, and several microbial-secreted volatile organic compounds (VOCs). We also provide only select examples of the effects of central carbon and nitrogen metabolism-linked metabolites on antibiotic susceptibility, as this topic has recently been reviewed and examined from a metabolic-reprogramming perspective (13). Of note, bacterial cell-to-cell communication mediated by acyl homoserine lactone quorum-sensing signals has been previously extensively reviewed and will not be discussed in this article (14–18). Additionally, we will highlight the progress made in the identification of novel potential drug targets and the discovery of antibiotic adjuvant compounds as a result of the mechanistic elucidation of chemical-mediated alteration of antimicrobial responses.

## CHEMICAL-MEDIATED ALTERATION OF ANTIBIOTIC SUSCEPTIBILITY

In this section, we will discuss different small molecules that elicit alteration of antibiotic susceptibility and the molecular basis thereof. We will attempt to identify novel targets that can be exploited for antimicrobial adjuvant therapy and progress made in identifying inhibitors of these targets (Table 1).

**TABLE 1:** Potential adjuvant targets to prevent small molecule-mediated AMR, tolerance, and persister formation

Target	Identified inhibitors, analogs, and delivery vectors	Potentiated antibiotics or *in vivo* observation	References
Polyamines			
Ornithine decarboxylase	Difluoromethylornithine Dicyclohexyamine	Polymyxin B (PMB)	(19–21)
SpeG	Diminazene Polyamine analogs, e.g., N1-Dodecylpropane-1,3-diamine (OES2-017)	Tetracycline, ciprofloxacin, vancomycin, kanamycin, and rifampicin.	(22, 23)
Polyamine uptake in alveolar macrophages	44-Ant-44 44-Bn-44	Prolongs survival of *Pneumocystis* pneumonia-infected rats.	(24)
Membrane and ribosome	Polyamine analogs – N1-Dodecylpropane-1,3-diamine (OES2-017) – Octan-1-amine (OES2-046) – (3-aminopropyl)-(octyl)amine (OES2-045) – Dioctylamine (OES2-008)	Azithromycin.	(25)
PQS			
PqsR	Quinazoline disulfide analog Quinazoline analog 3-hydroxy-pyridin-4(1*H*)-1 analog QSI-24 QSI-4 1H-Benzo[d]imidazole analog Psoralen	Tobramycin, PMB, levofloxacin, kanamycin, and ciprofloxacin *in vitro* and aminoglycosides *in vivo* in *C. elegans*.	(26–33)
PqsR/PqsBC	Nonligand-based benzamide–benzimidazole analog	Meropenem	(34)
PqsR/PqsD	Compound 6	Ciprofloxacin	(35)
PqsA	Norharmane 3-hydroxy-pyridinone analog	Carbapenems and PMB *in vitro* and polymyxin *in vivo* in a murine infection model.	(28, 36)
	PQS	Improved survival of a mouse model co-infected with *S. aureus* and *P. aeruginosa*.	(37)
PAA			
PaaE		Deletions/disruptions in *A. baumannii* and *B. cenocepacia* exhibit attenuated lethality *in vivo*.	(38–40)
PaaB		Deletions in *A. baumannii* exhibit decreased bacterial loads *in vivo* in a catheter urinary tract infection murine model and had increased sensitivity to ciprofloxacin, erythromycin, zeocin, and aminoglycosides *in vitro*.	(41)
PaaA		Insertion disruptions in *B. cenocepacia* exhibit a modest decreased lethality in a *C. elegans* infection model.	(38)
Indole			
TnaA	ALG-05 L-bishomotryptophan, S-phenylbenzoquinone-L-tryptophan, α-amino-2-(9,10-anthraquinone)-propanoic acid, L-tryptophan-ethyl ester, N-acetyl-L-tryptophan, S-phenylbenzoquinone-L-tryptophan, α-amino-2-(9,10-anthraquinone)-propanoic acid	Deletions in *E. coli* co-infecting *C. elegans* with *S*. Typhimurium and treated with ciprofloxacin exhibit a lower *S*. Typhimurium bacterial load.	(42–45)
Central carbon and nitrogen metabolism-linked metabolites			
Proton motive force	Pyruvate, inosine, fructose, glucose, mannitol, alanine, citrulline, and glutamine.	Aminoglycosides, tetracyclines, and β-lactam.	(46–57)
Porins OmpF, OmpK 36	Glutamine Inosine	β-lactams and tetracycline.	(55, 58)
Reactive oxygen species generation	Pyruvate Nitrite	Aminoglycosides and cefoperazone-sulbactam.	(59, 60)
VOC			
3-mercaptopyruvate sulfurtransferase	Aspartate	Ampicillin.	(61)
SseA		Deletions lead to lower *E. coli* bacterial loads *in vivo*.	(62)
Cystathionine β-synthase, cystathionine γ-lyase	DL-propargylglycine AOAA	Gentamicin, ampicillin, norfloxacin, and amikacin.	(61, 62)
Cystathionine γ-lyase	NL1	Gentamicin in *P. aeruginosa* and *S. aureus* murine infection.	(63)
	H_2_S sequestering delivery vector Gm@UiO-66-MA	Gentamicin.	(64)
	H_2_S scavenger 7B	Gentamicin.	(65)
	NO-containing implant devices	Reduce bacterial cell viability and disperse biofilms.	(66–68)
PurA	Aurodox	Aurodox has non-sufficient target selectivity. Δ*purA E. coli* exhibits colistin sensitivity.	(69, 70)

### Polyamines

The biogenic polyamines spermidine, spermine, putrescine, and cadaverine are small polycationic molecules ubiquitous in nature. The polyamine structure consists of primary and secondary amine groups spaced by hydrophobic hydrocarbon bridges. This unique structure of polyamines creates favorable interactions with macromolecules in living organisms, where they influence RNA transcription, chromosomal structure, protein synthesis, acid resistance, biofilm formation, signal cellular differentiation, and can scavenge for free radical ions (71, 72). Natural polyamines are involved in the host immune responses, inhibiting macrophage activation, inducing autophagy, and suppressing necrosis (73, 74). Spermine, spermidine, and putrescine are produced at higher levels by regenerating tissues and released by dying cells into the extracellular milieu at the infection site (73, 75), resulting in an accumulation of polyamines, reaching concentrations within the millimolar range, dependent on the infection location (75–78). Such polyamine accumulation was observed in *Pseudomonas aeruginosa* infections of the sinuses, lungs, and in the sputum of cystic fibrosis patients, as well as in *Staphylococcus aureus* abscess infections (75, 78). Another manifestation of the potential influx of polyamines at the infection site can be inferred from an iterative comparative metabolomics study of common gram-negative pathogens found in bloodstream infections (*Escherichia coli*, *Klebsiella* spp., and *Pseudomonas* spp.) that revealed that polyamines acetylated by polyamine acetyltransferases are elevated during infection (22).

#### Polyamine alteration of antibiotic responses

Several studies reported polyamine-mediated protection from antibiotic stress. Bactericidal antibiotics were shown to elicit oxidative stress, which may contribute in part to their lethality (79). One mechanism of the polyamine involvement in bacterial antibiotic stress response is alleviating the oxidative stress resultant from bactericidal antibiotic exposure (19, 80, 81). Endogenous polyamine biosynthesis increases in response to antibiotic-induced oxidative stress in bacteria, including *E. coli* and *Burkholderia cenocepacia*, where the promoters of genes encoding amino acid decarboxylases, namely ornithine decarboxylase (converting ornithine into putrescine) and lysine decarboxylase (producing cadaverine), are induced (19, 80, 81). Moreover, *Pseudomonas syringae* increases extracellular putrescine concentrations in response to H_2_O_2_ stress, and mutants that exhibit increased putrescine excretion have increased survival in the presence of H_2_O_2_ (82). However, *P. aeruginosa,* upon H_2_O_2_ stress, has conversely been shown to downregulate genes encoding polyamine biosynthesis enzymes (83). Exogenous supplementation of either putrescine or spermidine reduces intracellular reactive oxygen species (ROS) in *E. coli, B. cenocepacia,* and *P. syringae* (19, 81, 82, 84–87). Moreover, depriving bacteria of endogenous and exogenous polyamines, which can lead to growth defects, reduced their ability to cope with oxidative stress; for example, various polyamine biosynthesis deletion mutants of *E. coli* and *P. syringae* were rapidly killed by ROS when grown in polyamine-deplete media such as polyamine-free Vogel-Bonner medium (82, 85, 86, 88). Perturbation of putrescine synthesis in *B. cenocepacia* and spermidine synthesis in *P. aeruginosa* leads to an increase in ROS generation upon polymyxin B (PMB) stress (19, 89); an increase in membrane lipid peroxidation was further observed in *P. aeruginosa* (89). Furthermore, the deletion of spermidine synthesis in *Shigella* spp. results in a larger H_2_O_2_ zone of inhibition when grown on minimal media (90).

Polyamines alleviate oxidative stress through direct and indirect mechanisms. Spermine and spermidine can quench singlet oxygen, and all polyamines can scavenge hydroxyl and superoxide radicals (19, 91–93). Additionally, putrescine exerts its antioxidant protection effects by increasing the transcription of *rpoS* in *E. coli* and *oxyR* in *B. cenocepacia* and *E. coli,* and the overexpression of SoxR, RpoS, EmrR, and GshA in *E. coli* (86, 87, 94–97). RpoS is a general stress response regulator that responds to peroxides via induction of catalases KatE and KatG; genes encoding both were upregulated upon putrescine exposure (87). Additionally, micromolar concentrations of exogenous spermidine and putrescine induced the *rpoS* promoter and increased RpoS expression, which was shown to be responsible for an increase in persister cell formation in netilmicin-treated *E. coli* (95, 97). OxyR is a direct sensor of hydrogen peroxide stress that induces the expression of catalases and peroxidases, whereas SoxRS detoxifies superoxides via regulation of a >100 gene regulon that includes the superoxide dismutase SodA (98). Upregulation of GshA directly increases glutathione (GSH) synthesis, while EmrR, a negative transcriptional factor for multidrug efflux pumps, was hypothesized to indirectly increase GSH synthesis via inhibition of the efflux of GSH precursors (87). The resulting increase in GSH peroxidases detoxifies hydrogen peroxides (87). In contrast, excessive polyamines can promote toxic accumulation of superoxide radicals in *E. coli* and create improper cellular redox states in *Acinetobacter baumannii* (99–102). Polyamine-induced efflux of GSH was hypothesized to be a contributing factor to polyamine-β-lactam *in vitro* synergy against *A. baumannii* in checkerboard assays (101, 102). Together, polyamine homeostasis is fine-tuned in bacteria; the loss of biosynthesis or excess of polyamines can result in detrimental effects to the bacteria’s ability to manage oxidative stress and antibiotic insult.

Another mechanism of polyamine alteration of antibiotic susceptibility can be attributed to bacterial membrane effects. Membrane-level effects included changes to drug uptake and efflux, leading to polyamine-mediated protection from antibiotics. Polyamines have been shown to inhibit porin-mediated uptake of fluoroquinolone in *Mycobacteria* and *E. coli* and β-lactams in *E. coli* and *P. aeruginosa* (103–106). The modulation of porins was most pronounced by spermidine and spermine relative to other polyamines (103, 105, 106). Moreover, putrescine, spermidine, and cadaverine disrupt the interaction between the repressor AcrR and the *acrAB* promoter, resulting in induction of the multidrug efflux pump AcrAB-TolC (107). In *Burkholderia pseudomallei,* exogenous spermidine stimulated the *bpeA* promoter in promoter-reporter assays; BpeA is a component of the BpeAB-OprB efflux pump (108). In contrast, polyamine-mediated sensitization to antibiotics was observed, whereby increased levels of intracellular polyamines in *E. coli* led to aminoglycoside susceptibility by increasing the expression of OppA, a periplasmic protein involved in aminoglycoside uptake (109, 110).

Polyamine membrane effects also include their ability to perturb outer and inner membranes. Putrescine synergizes with macrolides in checkerboard assays against *Klebsiella pneumoniae* and *E. coli* via perturbation of the outer and inner membranes (25). Additionally, spermidine and spermine have been shown to displace Ca^2+^ from the outer membrane of *E. coli* to a similar extent as PMB nonapeptide (111). CRISPRi suppression of *speG,* encoding a polyamine acetyltransferase that detoxifies polyamines, in *E. coli* clinical isolates resulted in increased endogenous putrescine concentrations and decreased acetylated putrescine concentrations (22). These isolates exhibited increased outer and inner membrane permeability and increased intracellular antibiotic accumulation (22). However, it is unclear if this is a result of the heightened putrescine concentration, loss of acetylated putrescine, or other unrelated mechanisms (22).

**Fig 1:**
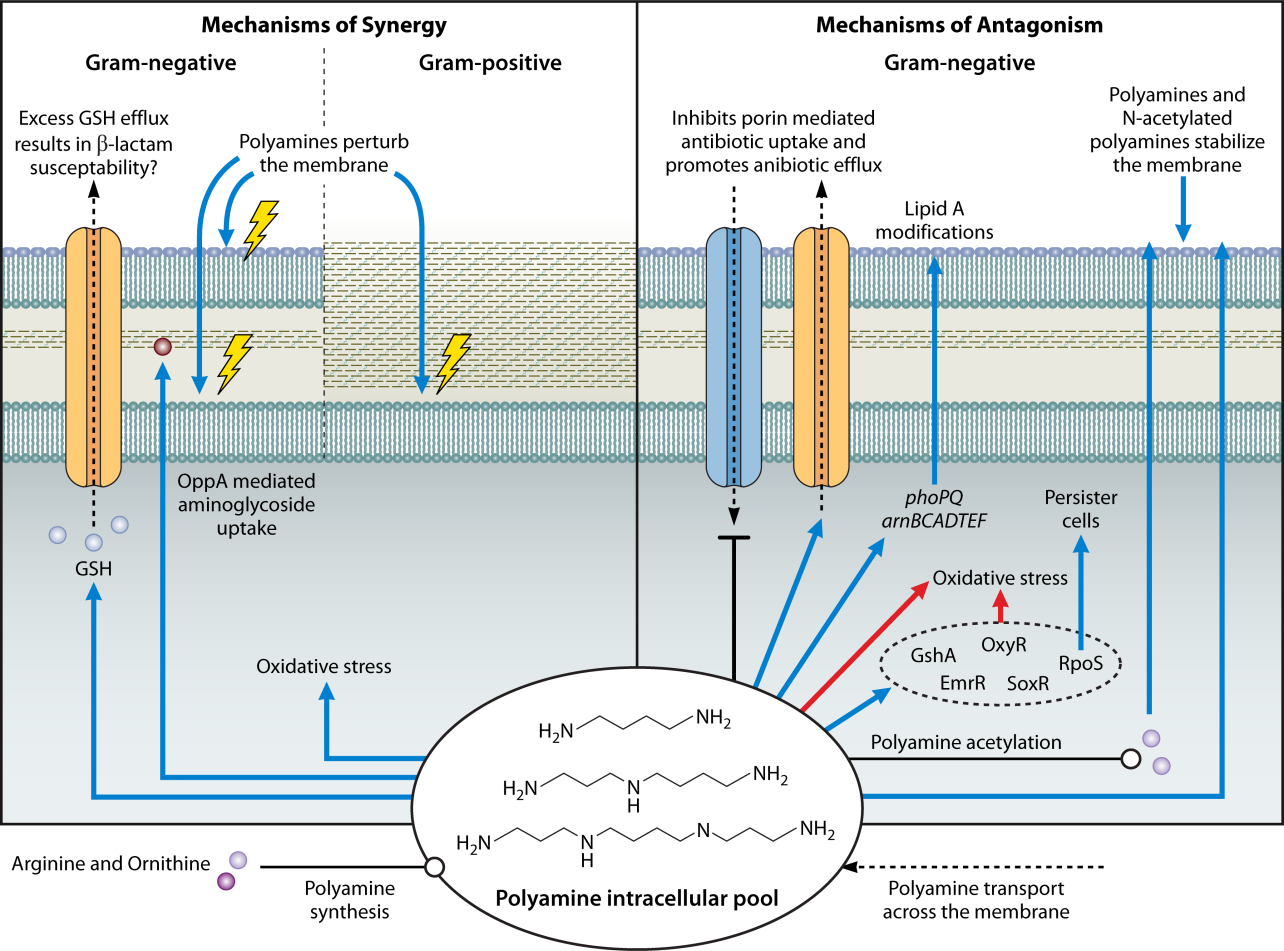
Mechanisms of polyamine-mediated alteration of antibiotic susceptibility. Polyamines synergize with antibiotics by increasing membrane permeability in both gram-negative and gram-positive bacteria. Synergy is also mediated by polyamine promotion of superoxide accumulation and creation of improper cellular redox states via polyamine-induced GSH efflux. Conversely, polyamines antagonize antibiotics in gram-negative bacteria via inhibition of porin-mediated antibiotic uptake, promoting antibiotic efflux pumps, inducing expression of lipid A modification pathways to combat polymyxins, alleviating oxidative stress, and promoting persister cell formation. Additionally, polyamines, and potentially acetylated polyamines, stabilize the outer membrane to reduce antibiotic permeability. The polyamine intracellular pool in bacteria consists of endogenously produced and exogenously acquired polyamines. For detailed descriptions of each mechanism and the corresponding references, refer to the text. Blue arrows denote increased, red arrows denote decreased, blunt-head arrows denote inhibited, dotted arrows denote the movement of a substrate, and open circled arrows denote synthesis.

Furthermore, polyamines can stabilize the outer membrane, increasing resistance to cationic peptides by up to 16-fold shift in the minimum inhibitory concentration (MIC) (19, 78, 112, 113). *P. aeruginosa* treated with spermidine exhibit induction of the two-component PhoPQ system and the *arnBCADTEF* operon, whose products mediate the addition of aminoarabinose to lipid A, a known mechanism for cationic peptide resistance in multiple gram-negative bacteria (113). Interestingly, exogenous polyamines also reduced cationic peptide-induced expression of *arnC*, suggesting that polyamines can compete with antimicrobial peptides for outer membrane binding (89). A membrane molecular dynamics simulation provided evidence that polyamines can stabilize the outer membrane via lipid A non-covalent crosslinks (114). Putrescine outcompeted PMB for surface binding on *B. cenocepacia*, contributing to putrescine-mediated protection from the antibiotic in wild-type and PMB-sensitive subpopulations of *B. cenocepacia* (19, 112). Polyamines at the infection site *in vivo* may also confer surface protection from cationic antimicrobial peptides; spermidine bound to the surface of *P. aeruginosa* wild type and Δ*pmrB*, shielding the membrane net negative charge and reducing the surface binding of PMB (78). *P. aeruginosa* lacking PmrB have impaired aminoarabinose modification of LPS, exhibiting an increased outer membrane negative charge and thus are more susceptible to PMB *in vitro;* however, in an *in vivo* sinus and lung murine infection model, these mutants do not exhibit bacterial load change when compared to the wild-type strain (78). The mechanisms of polyamine-mediated alteration to antibiotic susceptibility are summarized in Fig. 1.

#### Potential targets based on the molecular mechanism of polyamines

Interfering with polyamine homeostasis and environmental polyamine availability may provide new potential antimicrobial strategies. One potential strategy is the inhibition of polyamine synthesis. The inhibition of spermidine synthesis in *B. pseudomallei* via dicyclohexylamine, a bacterial and eukaryotic polyamine synthesis inhibitor (115, 116), exhibited a reduction in erythromycin efflux proposed to be due to downregulation of the efflux pumps BpeAB-OprB, AmrAB-OprB, and BpeEF-OprC (108). Dicyclohexylamine also inhibited the growth of *B. cenocepacia* by 75% at half-MIC of PMB, likely by reducing polyamine protection from PMB (112). Likewise, targeting polyamine synthesis using the ornithine decarboxylase inhibitor difluoromethylornithine is effective against the parasite *Trypanosoma brucei* subspecies and the fungus *Pneumocystis* pneumonia (PCP) (20, 21). Additionally, targeting the availability of polyamines in infection environments may serve as a novel strategy to prevent polyamine-induced protection from current therapies. Inhibitors, such as *N*-(4-amino-butyl)-*N*′-(10-{[4-(4-amino-butylamino)butylamino]-methyl}nthracene-9-ylmethyl)butane-1,4-diamine (44-Ant-44) and *N*-(4-amino-butyl)-*N*′-(4-{[4-(4-amino-butylamino)butylamino]-methyl}benzyl)butane-1,4-diamine (44-Bn-44), reduced polyamine uptake in alveolar macrophages, reducing environmental polyamine availability and prolonging the survival of PCP-infected rats (24).

Targeting polyamine detoxification mechanisms, such as the spermine/spermidine acetyltransferase SpeG, may serve as another target for antibiotic adjuvant therapies. CRISPRi suppression of *speG* in *E. coli* increases sensitivity by eightfold to vancomycin, an antibiotic typically only utilized against gram-positive bacteria due to its poor penetration of the outer membrane (22). Diminazene, a structural mimic of spermidine used as an antitrypanosomal and antibabesial agent and also shown to inhibit the human polyamine *N*-acetyltransferase SAT1 and bacterial SpeG in *E. coli* (22, 117), increased the sensitivity of *E. coli* to vancomycin eightfold (22). Diminazene also re-sensitized multidrug-resistant *E. coli*, *K. pneumoniae*, and *P. aeruginosa* to tetracycline and ciprofloxacin in checkerboard assays *in vitro* and synergized with tetracycline *in vivo* in an *E. coli* cecal-slurry model of sepsis by increasing intracellular accumulation of the antibiotics (22). Recently, a polyamine analog has been identified as a novel broad-spectrum inhibitor of SpeG in *S. aureus* (SpeG^+^ strains) and other various gram-negative and gram-positive pathogens (23). This inhibitor, acting at low micromolar ranges, synergized with polyamines, lowering their bacterial growth inhibitory concentrations by 32-fold, abolished polyamine-mediated resistance to the antibiotics vancomycin, kanamycin, and rifampicin *in vitro*, and eradicated SpeG-expressing *Salmonella enterica* serovar Typhimurium inside murine macrophages (23). However, the utility of SpeG as an adjuvant target may be species and strain specific. Despite generally being non-essential for bacterial survival, SpeG was essential in two *E. coli* clinical isolates but not in other *E. coli* strains such as the non-pathogenic model strain BW25113 (22). Moreover, *in silico* analyses on *speG* in *Shigella* spp. predicted that all analyzed genome sequences harbored inactivating mutations within the *ynfB-speG* operon and a diverse panel of *Shigella* spp. exhibited over a twofold increase in endogenous spermidine when compared to *E. coli* (90). The increase in endogenous spermidine improved *Shigella flexneri* fitness in the adverse environment of murine macrophages (90). As such, it is conceivable that targeting SpeG would only be advantageous against bacteria with the intact enzyme. Additionally, targeting SpeG in backgrounds in which it is essential would be lethal with increased selective pressure for resistance development.

Similarly, polyamine analogs have been shown to sensitize *K. pneumoniae* and *S. aureus* to azithromycin (by up to 32-fold), mimicking the mechanism of natural polyamine-azithromycin synergy but at substantially lower concentrations (23, 25). Together, the effects of polyamines on bacterial responses to antibiotics provide a promising strategy to identify novel potential targets to combat AMR (Table 1). Nonetheless, the redundancy of polyamine biosynthesis pathways and the functional and, to some extent, structural homology between the bacterial and human enzymes in these pathways present challenges that will need to be addressed to selectively and effectively exploit these targets for antibacterial development. Further research into the identification of new inhibitors that are specific to targeting bacterial enzymes is needed to better quantify the therapeutic potential of targeting polyamine-related mechanisms.

### PQS

PQS or 2-heptyl-3-hydroxy-4-quinolone is a quorum-sensing compound that mediates the production of numerous virulence factors and induces biofilm formation (118). PQS production is regulated by PqsR, a LysR-type transcriptional regulator that integrates PQS signaling into the broader quorum-sensing network, yet this molecule also exhibits non-quorum-sensing properties, which have been previously reviewed in reference 118. PQS controls the expression of 182 genes; 179 of those genes are controlled via PqsR-independent pathways, highlighting its extensive non-quorum-sensing role (119). Notably, sputum samples from cystic fibrosis patients infected with *P. aeruginosa* contain PQS in the nanogram/milliliter range, and clinical isolates from the sputum of young children exhibited a relative increase in PQS production compared to isolates taken from blood and urinary infections when cultured in a rich medium (120–122). These results suggest that PQS plays an important role in the long-term persistence of infection.

#### PQS alteration of antibiotic responses

PQS plays a role in bacterial antibiotic responses, where altering PQS levels, whether exogenously available or endogenously produced, has been shown to influence the antibiotic susceptibility of *P. aeruginosa*. Exogenous PQS sensitizes *P. aeruginosa* PAO1 to chloramphenicol, β-lactams, tetracycline, and aminoglycosides in disc diffusion assays (123). Furthermore, exogenous PQS and its endogenous overproduction by disrupting *pqsL* in PAO1 also increased susceptibility to fluoroquinolones (124). The loss of endogenous PQS by deletions in the PQS biosynthetic pathway (*P. aeruginosa* PAO1 Δ*pqsH*) results in increased resistance to aminoglycosides (by at least a twofold MIC shift) (125). A Δ*pqsA* mutant that exhibits loss of PQS production also exhibits increased tolerance, as determined by a higher CFU count after 3 hours of treatment, to fluoroquinolones and aminoglycosides (124). Additionally, a *P. aeruginosa* PAO1 small colony variant resistant to an aminoglycoside showed significant reduction in PQS production and *mexGHI-opmD* transcription (126). The deletion of *mexI* and *opmD* in PAO1 led to reduced endogenous PQS concentrations and resistance to fluoroquinolones and aminoglycosides in disk diffusion assays, similar to *P. aeruginosa* PAO1 Δ*pqsH* or Δ*pqsA* (123). Exogenous supplementation of PQS re-sensitized the Δ*mexI* and Δ*opmD* mutants to the antibiotics, as did the genetic complementation (123). These results further support PQS as a synergist of the above-mentioned antibiotics. However, additional studies were unable to reproduce the previously shown decrease in PQS production in *mexGHI-opmD* mutants of PAO1 and PA14 (127, 128). The discrepancies in these results may have arisen from condition-dependent effects (e.g., detection of PQS from liquid [123] vs solid [127] cultures). Nonetheless, *P. aeruginosa* appeared to lower its production of PQS in response to certain antibiotics, where microarray data of PAO1 showed that PQS biosynthesis genes (*pqsA*, *pqsB*, *pqsC*, *pqsD*, *pqsE*, and *pqsH*) are repressed upon exposure to sub-MIC concentrations of azithromycin, β-lactams, and fluoroquinolones (129). Conversely, *P. aeruginosa* PA14 treated with β-lactams exhibits no alteration in PQS production or accumulation (125). Both *P. aeruginosa* PA14 and PAO1 produce similar levels of PQS, but PAO1 PQS is mostly cell-associated (72%), whereas a majority of PA14 PQS (78%) is extracellular; thus, PA14 may not need to lower PQS production to mitigate PQS stress intracellularly (130). Notably, PQS may act as an endogenous stressor, evident from the ability of high levels of endogenously produced or exogenously added PQS to increase bacterial autolysis and sensitivity to antibiotics (123–125, 131).

Multiple mechanisms may underlie the ability of PQS to synergize with the above-mentioned antibiotics. One could be through the pro-oxidant activities of PQS (124, 132). It is probable, similar to polyamines, that PQS concentrations above their homeostatic range result in oxidative stress, increasing bacterial sensitivity to stressful conditions (124, 125, 133). Moreover, PQS sensitization to antibiotics could be through membrane alteration, reducing the membrane barrier effect for certain antibiotics. PA0011 in *P. aeruginosa* PAO1 encodes a 2-OH-lauroytransferase involved in lipid A biosynthesis and is negatively regulated by PQS (134). *P. aeruginosa* mutants with insertional inactivation of PA0011 exhibit a twofold or greater decrease in the MIC of fluoroquinolones, tetracycline, chloramphenicol, polymyxin, rifampicin, and β-lactams (134). *P. aeruginosa* strains deficient in PQS biosynthesis (Δ*pqsR* and Δ*pqsH*) exhibited increased PA0011 expression (134).

Conversely, PQS may improve the bacterial response to certain antibiotics. One example is that PQS production was also related to conferring carbapenem tolerance (135). Deletion of *rpoN*, an alternative sigma factor, upregulated *pqsA*, *pqsH*, and *pqsR*, increasing PQS production, and led to carbapenem tolerance in *P. aeruginosa* PAO1 (135). Exogenous PQS decreased bacterial killing by over 90% by the carbapenem biapenem in the *P. aeruginosa* wild-type strain, and the Δ*rpoN* Δ*pqsA* exhibited a loss of the carbapenem-tolerant phenotype, demonstrating that the carbapenem resistance phenotype in the Δ*rpoN* background is mediated by increased PQS (135). VqsR is involved in carbapenem tolerance in stationary phase *P. aeruginosa* cells, and *vqsR* knockout mutants exhibit higher bacterial cell death upon carbapenem treatment; the expression of VqsR is under the control of RpoN and PQS (136). Additionally, *P. aeruginosa* Δ*ptrA* exhibits downregulation of PQS biosynthesis genes and a twofold decrease in the MIC of carbapenem (137).

The effect of PQS on susceptibility to other antibiotics, namely cationic antimicrobial peptides, is yet to be fully determined. The supernatant of subinhibitory colistin-treated *P. aeruginosa* PAO1 cultures shows increased PQS extracellular concentrations (138), yet *P. aeruginosa* PAO1 Δ*pqsR* mutants, which exhibit downregulated PQS synthesis genes, are PMB tolerant in bacterial time-kill curves (139).

PQS-mediated protection from antibiotics such as biapenem could be due, in part, to its effects on outer membrane vesicle (OMV) formation. PQS is trafficked between cells in OMVs, and the level of OMV formation is closely aligned with the level of PQS endogenously produced (130, 140–143). OMVs have been previously shown to sequester membrane-acting antimicrobials and carbapenems (144, 145), possibly contributing to increased resistance to these antibiotics, which would align with the observed increase in PQS synthesis by membrane-acting compounds (138). Interestingly, exogenous PQS is capable of forming OMVs not only in *P. aeruginosa* but also in *E. coli* and *K. pneumoniae* (142), suggesting its effects are not restricted to *P. aeruginosa* but extend to other bacterial species. These observations warrant further investigation into the effects of PQS on non-producers.

**Fig 2:**
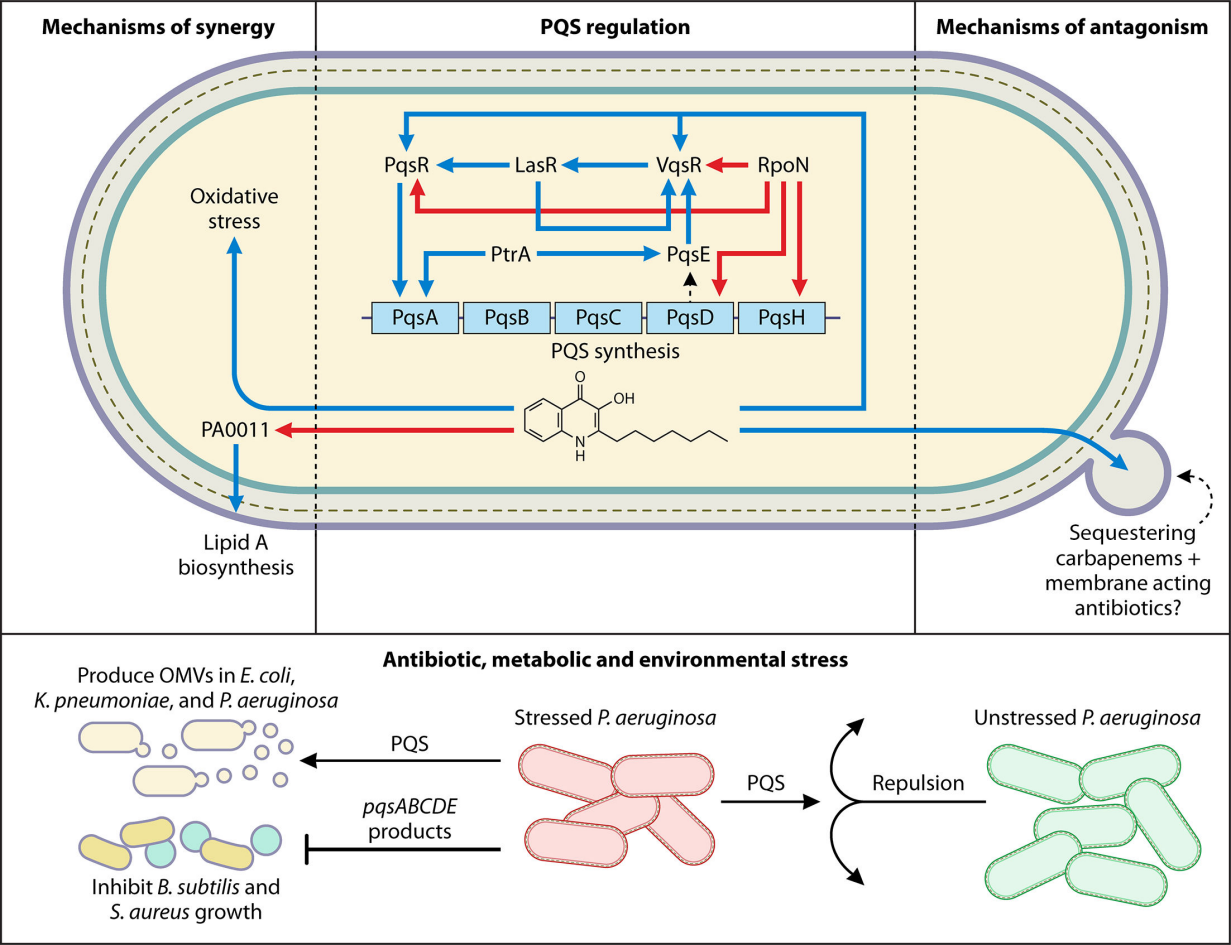
Mechanisms of PQS-mediated alteration of antibiotic susceptibility. PQS synergizes with antibiotics by promoting oxidative stress and decreasing lipid A biosynthesis via downregulation of PA0011. Conversely, PQS could be antagonizing membrane-acting antibiotics by sequestering antibiotics with OMVs. PQS also impacts the surrounding bacterial population, where PQS secretion is upregulated in stressed *P. aeruginosa* populations to act as a long-range signal to repulse unstressed *P. aeruginosa* populations. Additionally, PQS can induce OMV production in other gram-negative bacteria, and the products from the pqsABCDH operon can inhibit gram-positive bacterial growth. Descriptions of each mechanism and the corresponding references are detailed in the text. Blue arrows denote increased, red arrows denote decreased, blunt-head arrows denote inhibited, dotted arrows denote the movement of a substrate, and open circled arrows denote synthesis.

PQS plays a role in shaping bacterial populations in monoculture and polymicrobial communities. Interestingly, there was a concentration-dependent increase in PQS concentrations, detected by surface density, in aminoglycoside-treated *P. aeruginosa* PA14 swarming communities (125). PQS increased *P. aeruginosa* susceptibility to aminoglycosides as mentioned above. Yet, it was hypothesized that PQS may have an essential role in combating aminoglycoside stress by acting as a long-range life and death signal (124, 125, 146). The heightened PQS concentrations in response to aminoglycosides may serve as an attempt to prevent swarm expansion into the antibiotic-containing region, repelling approaching *P. aeruginosa* swarms far beyond that area (125, 146). This would suggest that PQS stress response is also a coordinator of spatial organization during swarming and is a collective stress response for the entire population (146). *P. aeruginosa* inoculated on semisolid agar in proximity to *E. coli* showed that *P. aeruginosa* steadily expanded toward *E. coli* and accelerated the production of PQS, which was detected by 48 h compared to 96 h in *P. aeruginosa* monoculture (147), suggesting that PQS has a spatiotemporal distribution that may garner an adaptive advantage for *P. aeruginosa* within its polymicrobial environment (147). PQS also has growth inhibitory activity against *S. aureus* and *Bacillus subtilis,* where total organic extracts from PA14 wild type but not a *pqsA*^–^ mutant inhibited the growth of both gram-positive species (148). PQS inhibited the motility of various gram-positive (e.g., *B. subtilis*, *S. aureus*, and *Staphylococcus epidermidis*) and gram-negative (e.g., *E. coli* and *Proteus vulgaris*) bacteria in semi-solid motility assays (149). Culture supernatants from several *P. aeruginosa* isolates stimulated biofilm formation in *S. aureus*, an effect positively correlated to the levels of PQS produced by the *P. aeruginosa* isolates and significantly reduced in PQS-deficient mutants (150). Together, these examples and others (reviewed in reference 118) show that PQS can lead to changes in biodiversity, richness, and structure of the microbial community. Together, PQS can both contribute to or respond to stress, depending on the context and type of antibiotic, and can help shape microbial communities, potentially conferring an advantage to *P. aeruginosa* in polymicrobial populations. Figure 2 summarizes the mechanisms of PQS-mediated alteration to antibiotic susceptibility.

#### Potential targets based on PQS alteration of antibiotic susceptibility

PQS has a complex role in shaping *P. aeruginosa* environments and in mediating stress responses that is not yet fully understood, but the elucidation of PQS mechanisms has shed light on multiple potential drug targets (Table 1). Interestingly, PQS itself has been shown to exhibit potential promise as an antibacterial, where PQS improved the survival of mice co-infected with *S. aureus* and *P. aeruginosa* (37). Potential analogs of PQS could work mechanistically similar to that of natural PQS, inducing antibiotic sensitivity, but at substantially lower concentrations, similar to the strategy employing polyamine analogs described above (25). Although PQS was shown to increase *P. aeruginosa* susceptibility to tobramycin, it was overexpressed in response to tobramycin treatment, suggesting PQS may serve an important role for survival of the population. As such, PqsR is a potential target due to its positive feedback effect on PQS production. Quinazoline disulfide analogs, quinazoline analogs, 3-hydroxy-pyridinone analogs, and other PqsR inhibitors (see Table 1) have been shown to potentiate tobramycin in *P. aeruginosa*-infected murine models, lowering cell viability of both *S. aureus* and *P. aeruginosa* in a mixed biofilm and of *P. aeruginosa* biofilms (26–30). Although exogenously supplemented PQS was shown to confer antibiotic susceptibility to fluoroquinolones, aminoglycosides, and tetracyclines, inhibitors of PqsR and PqsR/PqsD synergized with fluoroquinolones and aminoglycosides against planktonic cells, with fluoroquinolones and tetracycline against *P. aeruginosa* biofilms, and with aminoglycosides against *P. aeruginosa* in *Caenorhabditis elegans* infection assays (Table 1) (26, 28, 31–33, 35). Moreover, targeting PQS biosynthesis could lower PQS-induced protection to carbapenems and polymyxin. PqsA inhibitors norharmane and a 3-hydroxy-pyridinone analog each synergized with polymyxin to treat *P. aeruginosa* planktonic cells and a norharmane-polymyxin combination yielded lower bacterial burden in a murine lung infection model (Table 1) (28, 36). Additionally, norharmane and a dual PqsR/PqsBC inhibitor (a benzamide-benzimidazole analog) each synergized with carbapenem to treat *P. aeruginosa* planktonic cells (34, 36).

### PAA

PAA is an intermediate by-product in the degradation of aromatic amino acids, yielding the common metabolic intermediates succinyl-CoA and acetyl-CoA (151). The operon *paaABCDEFGHJKXYI* encodes the enzymatic pathway for PAA catabolism and is one of the most differentially regulated pathways in bacteria in response to environmental stimuli (152–156). PAA is a known signaling compound in plants, acting similarly to an auxin, regulating plant growth and development (157); auxins are known to have regulatory effects on surrounding soil bacteria (158). PAA is also detected in humans and is predominantly derived from gut microbiota-dependent metabolic conversions (159); hence, bacteria may encounter PAA during infection. PAA and its catabolism have been implicated in bacterial pathogenesis in several infection models (38, 41, 160–162). In an *A. baumannii* zebrafish infection model, PAA injection into zebrafish otic vesicles (as little as 3.4 ng) or increased PAA excretion due to the loss of bacterial PAA catabolism via Δ*paaA* or Δ*gacS* (positive regulator of the *paa* operon) enhanced neutrophil migration and clustering at the site of infection (39, 160), ultimately leading to greater bacterial killing and improved host survival (160). Blocking PAA catabolism by deleting *paaB* also resulted in attenuated virulence of *A. baumannii* in a murine catheter-associated urinary tract infection model (41). Similarly, PAA catabolism was required for the pathogenicity of *B. cenocepacia* in *C. elegans* (38), whereas *P. aeruginosa*-infected *C. elegans* exhibited increased survival upon 200 µg/mL PAA treatment (161). However, *C. elegans* infected with *S. aureus*, *S. epidermidis* (PAA non-catabolizing pathogens), and *B. cenocepacia* did not have altered survival rates upon PAA treatment, but a *B. cenocepacia* Δ*paaABCDE* mutant treated with PAA did exhibit increased *C. elegans* survival (162). Furthermore, genes specifically associated with dominant clonal clusters of *Mycobacteroides abscessus* infecting cystic fibrosis patients were enriched for PAA catabolism, pointing to the potential infection relevance of PAA (163). Together, PAA acts as a regulatory signal that bacteria may encounter during infection, which could contribute to establishing persistent infections and responding to antibiotic stress.

#### PAA alteration of antibiotic responses

**Fig 3:**
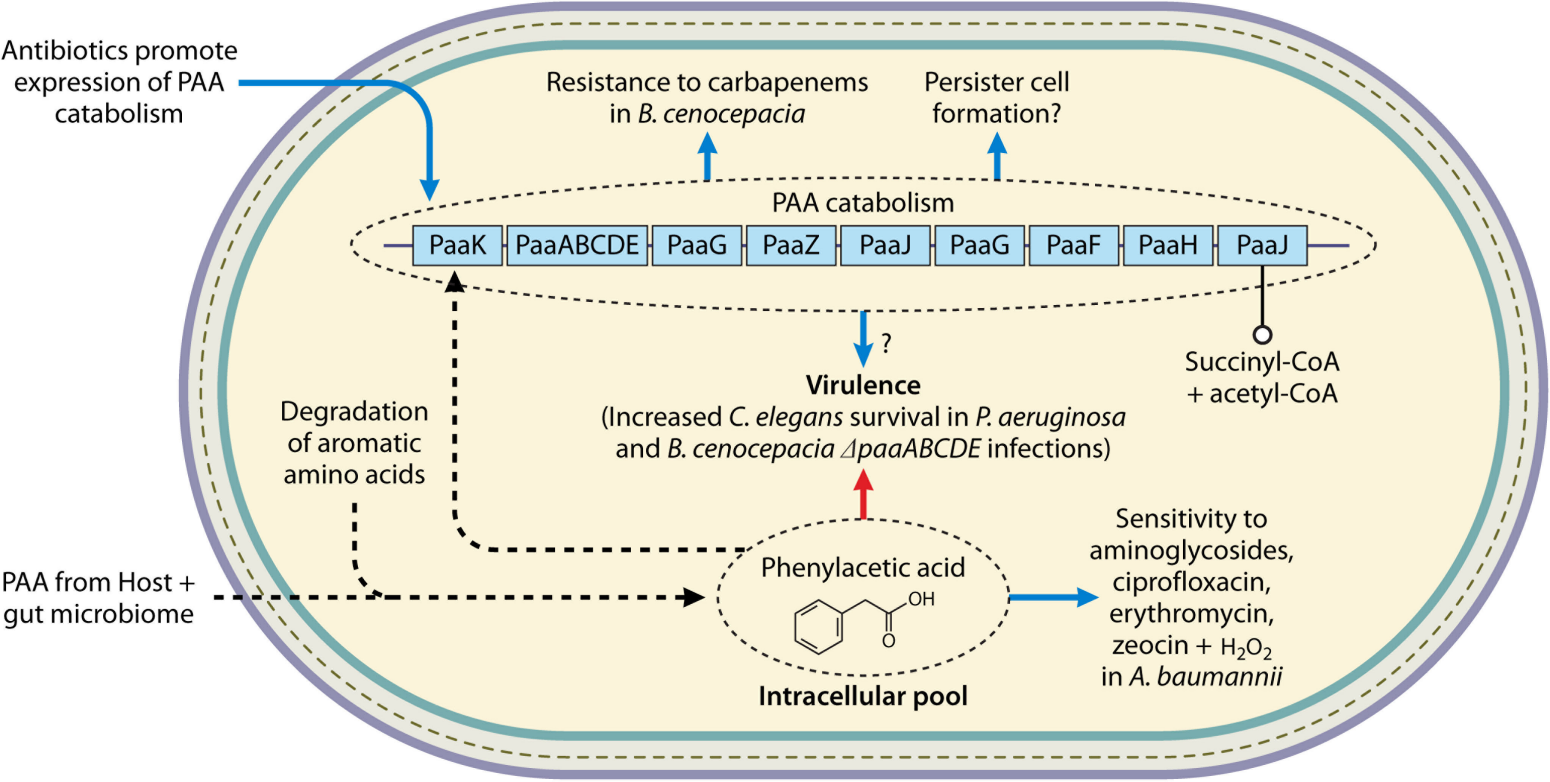
Mechanisms of PAA-related alteration of antibiotic susceptibility. In gram-negative bacteria, promoting PAA accumulation, through either the inhibition of catabolism or uptake of exogenous PAA, leads to increased sensitivity to aminoglycosides and H_2_O_2_ and may decrease virulence. PAA degradation has been shown to be positively regulated by antibiotic exposure, and PAA plays a role in carbapenem resistance and persister cell formation. For detailed descriptions of each mechanism and the corresponding references, refer to the text. Blue arrows denote increased, dotted arrows denote the movement of a substrate, and open circled arrows denote catabolism.

PAA catabolism may contribute to bacterial response to antibiotic and oxidative stress. The *paa* operon is highly expressed in *A. baumannii* persister cells induced by aminoglycosides, β-lactams, PMB, tetracycline, and fluoroquinolones (154, 164). Likewise, *A. baumannii* upregulates the *paa* operon upon treatment with trimethoprim-sulfamethoxazole (41). Knockouts of *paaB* in *A. baumannii* Ab17978 and the pathogenic UPAB1 strains have increased endogenous levels of PAA, coinciding with a twofold decrease in ciprofloxacin, erythromycin, and zeocin MIC in both strains, with the latter exhibiting a 2-log reduction in bacterial survival when exposed to H_2_O_2_ relative to the parent strains (41). Interestingly, *A. baumannii* UPAB1 *ΔpaaB* exhibits a fourfold decrease in aminoglycoside MIC, while *A. baumannii* Ab17978 *ΔpaaB*, whose wild type is already fourfold more susceptible to aminoglycosides than UPAB1, exhibits no further change in MIC but does exhibit a reduction in growth under aminoglycoside treatment (41). Moreover, PAA degradation played a role in spontaneous resistance to carbapenems in *B. cenocepacia* (165). The mechanisms of PAA-related alteration to antibiotic susceptibility are summarized in Fig. 3.

#### Potential targets from PAA alteration of antibiotic susceptibility

To date, there are no known inhibitors that have been directly confirmed to target proteins encoded by the *paa* operon. However, targeting the proteins early in the PAA metabolic pathway may serve as viable drug targets to enhance susceptibility to aminoglycoside (and possibly other antibiotics mentioned above) and increase bacterial killing through neutrophil recruitment (Table 1) (41, 160). Analogs of PAA could potentially serve as antibiotic adjuvants, acting similarly to the natural PAA. The PAA analog diclofenac, a non-steroidal anti-inflammatory, has antimicrobial activity and potentiates streptomycin, an aminoglycoside, against *E. coli* and *S. aureus* in checkerboard assays, potentiates colistin against *K. pneumoniae*, *P. aeruginosa,* and *A. baumannii in vitro* and against *A. baumannii in vivo* in a murine model of pneumonia (166, 167). The increased aminoglycoside sensitivity was shown to be a result of inhibition of bacterial DNA synthesis by diclofenac, and the increased colistin sensitivity was largely attributed to a decrease in type IV pili, but repression of PAA catabolism was also observed (166, 167). Additionally, targeting PAA catabolic enzymes could attenuate virulence; the phenotypes of *paa* gene deletion mutant phenotypes of *B. cenocepacia, A. baumannii,* and *P. aeruginosa* in *in vivo* models provide proof-of-concept for such an approach (38–41, 161). The role of PAA as a signaling molecule within the microbial community, its impact on antibiotic susceptibility, and the identification of inhibitors require further research.

### Indole

Indole is an aromatic heterocyclic organic compound produced via the tryptophanase TnaA from tryptophan by multiple gram-negative and gram-positive bacteria and was shown to influence antibiotic resistance and persistence (168–170). The physiologically relevant concentration of indole varies across the bacterial growth cycles; for example, *E. coli* cells produce indole bursts (>60 mM) when entering the stationary phase, with indole concentrations subsiding soon after (171); this form of indole signaling is termed pulse signaling and is only experienced by the producer cell (172). The growth medium of *E. coli* grown into the stationary phase contains approximately 0.5–1.0 mM, whereas *in vivo* concentrations vary across body sites and individuals, where human stool samples range from 0.3 to 6.64 mM and plasma concentrations from 5.12 to 72.6 nM (172–174). These indole concentration ranges are typically considered physiologically relevant for long-term exposure; such persistent signaling can be experienced by producer and non-producer cells (172).

#### Indole alteration of antibiotic responses

Indole has been both shown to inhibit and promote persister formation, depending on the experimental design, including the antibiotic-indole combinations and concentrations and timing of indole exposure, as reviewed by Zarkan et al. (172). New research has since provided evidence of indole pulse signaling being responsible for the formation of quinolone *E. coli* persisters (171, 175). An artificial pulse of indole in quinolone-treated *E. coli* Δ*tnaA* (indole-negative) led to an increase in quinolone persister cells. In the absence of exogenous indole, this mutant exhibited a 2–10-fold decrease in quinolone persister formation compared to the wild type (175). Quinolones inhibit DNA gyrase binding to gyrase A; indole binds to gyrase B, an interaction hypothesized to protect against quinolones while reducing DNA gyrase activity, inhibiting DNA replication, and inducing a dormant state (175, 176). Notably, indole appeared to confer protection against fluoroquinolone *in vivo,* where *C. elegans* co-infected with *E. coli* Δ*tnaA* and *S*. Typhimurium in an intestine infection model had a lower *S*. Typhimurium bacterial load compared to a co-infection with indole-producing wild-type *E. coli* after fluoroquinolone treatment (42). Indole does not have an effect on novobiocin persister formation, an expected result given that they both interact with gyrase B (175). Moreover, indole is produced by *Vibrio cholerae* in response to sub-MIC aminoglycosides, and the exogenous addition of indole during near-exponential phase culture increases persistence to aminoglycosides (177). Indole induces the expression of the ribosome-associated factor RaiA*,* which is proposed to result in the preservation of non-translating inactive but intact ribosomes that could be rapidly reactivated upon stress relief, conferring a survival advantage (177). Interestingly, indole does not have an effect on *V. cholerae* persistence to carbenicillin or trimethoprim (177). In contrast, *E. coli* Δ*tnaA* showed an increase in ampicillin (β-lactam) persisters during the exponential phase relative to the wild type; however, the opposite was observed during the stationary phase (178). *E. coli* persisters that survive ampicillin treatment display a lower intracellular pH and a tighter grouping of pH within the persister population (178). Conversely, *E. coli* Δ*tnaA* exhibits a higher cytoplasmic pH, and artificial pulsing restores the pH to that of the wild type (178, 179). These examples suggest that the effects of indole on persister formation may be antibiotic and growth-phase-specific. However, some of the disparities in the reported effects of indole on bacteria may have stemmed from effects related to different solvents used to solubilize indole (e.g., DMSO vs ethanol; reviewed by Song and Wood [180]). Furthermore, potential discrepancies between expected outcomes of bacterial exposure to exogenous indole and the phenotypes of a ∆*tnaA* strain may stem from the fact that the mutant not only lacks endogenous indole production but also accumulates tryptophan and lacks Tna-dependent pyruvate production, which together may confound the indole-related results. These variables should also be considered when interpreting the findings and comparing the different studies.

In addition to its role in persister formation, indole alters the susceptibility of multiple gram-negative bacteria to antibiotics. Indole is overproduced during sub-MIC aminoglycoside exposure in *V. cholerae* and is secreted by *E. coli* spontaneous drug-resistant mutants, shielding sensitive strains from antibiotic insult (177, 181). Additionally, exogenous indole increases antibiotic resistance in *E. coli*, *K. pneumoniae*, *P. aeruginosa,* and *S*. Typhimurium as determined *in vitro* by CFU counting and disk diffusion assays (42, 182–185). Indole-mediated alterations of antibiotic susceptibility are associated with its effects on efflux pump activation; these have been previously reviewed (172), hence will not be detailed herein. Conversely, indole from donor *E. coli* extracts was more recently shown to sensitize *P. aeruginosa, Proteus mirabilis, and K. pneumoniae* clinical isolates to fluoroquinolones, β-lactams, and aminoglycosides (186). qRT-PCR analyses concluded that indole downregulated genes encoding RND-type efflux in *K. pneumoniae* (186). On the other hand, indole has recently been shown to facilitate the uptake of the antimicrobial quaternary ammonium cation, malachite green, in *E. coli* and *P. aeruginosa* (187). Time-resolved second-harmonic light scattering showed that micromolar concentrations of indole increased the rate of MG transportation across the bacterial cytoplasmic membrane via an Mtr permease and enhanced passive diffusion (187).

**Fig 4:**
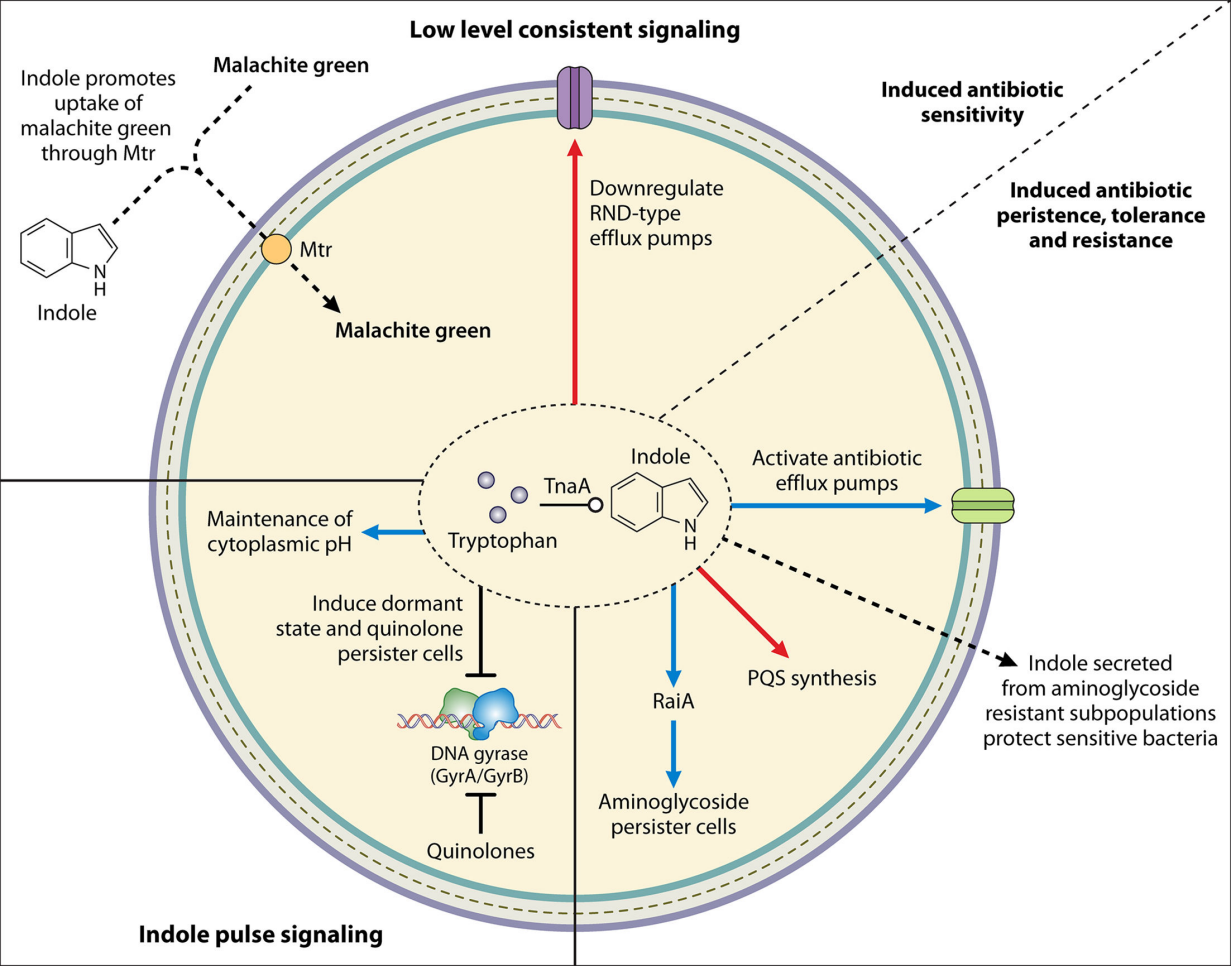
Mechanisms of indole-mediated alteration of antibiotic susceptibility. Indole has two forms of signaling, a low-level consistent signaling (persistent signaling) where bacteria are consistently exposed to 0.005–6.64 mM of indole, and pulse signaling, in which bacteria are temporarily exposed to indole concentrations of up to 60 mM. Low-level consistent signaling can synergize antibiotics by downregulating RND-type efflux pumps and has been shown to promote malachite green uptake, increasing transportation across the membrane. Conversely, low-level consistent signaling can antagonize antibiotics by activating efflux pumps and inhibiting PQS synthesis in *P. aeruginosa* and promote persister cell formation in *V. cholerae* via inducing the expression of RaiA. Indole has also been shown to be secreted from aminoglycoside-resistant sub-populations of *E. coli* and protect some of the surrounding sensitive population. Indole pulse signaling has been shown to inhibit GyrB and promote quinolone persister cell formation and to be important for the maintenance of cytoplasmic pH. For detailed descriptions of each mechanism and the corresponding references, refer to the text. Blue arrows denote increased, red arrows denote decreased, blunt-head arrows denote inhibited, dotted arrows denote the movement of a substrate, black flat-headed arrows denote inhibition, and open circled arrows denote synthesis.

Interestingly, indole signaling may overlap with PQS signaling in *P. aeruginosa*. Indole was shown to induce *P. aeruginosa* PAO1 resistance to tetracycline, aminoglycosides, and β-lactams *in vitro* by repression of *mexGHI-opmD* and PQS synthesis genes (185). Indole supplementation does not further decrease PQS concentrations in a *pqsR* knockout mutant, suggesting that indole signaling is linked to PQS via *pqsR* suppression (185). Resistance to aminoglycosides and tetracycline matches previous reports discussed under the PQS alteration of antibiotic susceptibility section (123, 125, 185). The mechanisms of indole-mediated alteration to antibiotic susceptibility are summarized in Fig. 4.

#### Potential targets based on indole alteration of antibiotic susceptibility

Indole appears to be mediating variable effects on bacterial responses to antibiotics, and more conclusive results may be needed; nonetheless, TnaA represents a viable antibiotic adjuvant drug target to mitigate indole-mediated antibiotic resistance and persister formation. Multiple TnaA inhibitors have been identified, such as ALG-05, and shown to reduce cecal indole and serum indoxyl sulfate, a product of microbial tryptophanase, *in vivo* and inhibit indole production by *E. coli in vitro* (Table 1) (43–45). However, these compounds have yet to be tested in combination with antibiotics. Furthermore, indole derivatives have been shown to eradicate *E. coli*, *S. aureus, P. aeruginosa, S. epidermidis, Enterobacter tabaci,* and *Mycobacterium tuberculosis* persister cells alone and in combination with antibiotics (recently reviewed in reference 180).

### Central carbon and nitrogen metabolism-linked metabolites

Due to the high interconnection of metabolic pathways, the environmental scarcity or abundance of metabolites can alter the metabolic states of bacteria, whereby key metabolites can activate slowed metabolisms, which is a characteristic of antibiotic-tolerant and persister cells (13, 188). Hence, the abundance of host- or bacterial-derived metabolite intermediates from the central carbon and nitrogen metabolism, including but not limited to pyruvate, succinate, glutamine, and alanine (see Table 1 for the full list), can activate metabolisms, promoting bacterial killing by bactericidal antibiotics (recently reviewed in reference 13). Similarly, the host immunometabolite itaconate, which is derived from the tricarboxylic acid cycle intermediate *cis*-aconitate, can be used as a carbon source by *P. aeruginosa* (189, 190). Additionally, itaconate has been shown to alter *P. aeruginosa* virulence and antibiotic susceptibility (190–192). Host itaconate, glutamate, and succinate have been shown in *ex vivo* and *in vivo* models to be secreted at high enough concentrations to alter antibiotic susceptibility and virulence (190–193). This topic has been recently reviewed in depth by Peng et al. (13) through the context of metabolic reprogramming; hence, this section will provide only select examples of the effects of central carbon and nitrogen metabolism-linked metabolites on antibiotic susceptibility.

#### Central carbon and nitrogen metabolism-linked metabolite-mediated alteration of antibiotic responses

Allison and colleagues (46) showed that supplementing carbon sources that enter upper glycolysis, as well as pyruvate, with aminoglycosides increased bacterial killing of *E. coli* and *S. aureus* persister cells. These metabolites activate central metabolism and the electron transport chain, which elevates the bacterial proton motive force (PMF), increasing aminoglycoside uptake (46). Similar observations were reported with aminoglycoside-resistant *Edwardsella tarda*, *Salmonella* spp., *P. aeruginosa*, *K. pneumoniae*, *Vibrio alginolyticus*, and *Vibrio parahaemolyticus* (47–54). Similarly, pyruvate, inosine, and fructose-driven increases in PMF have been shown to promote tetracycline uptake in tetracycline-resistant *E. coli* and *K. pneumoniae*, ampicillin uptake in *Streptococcus agalactiae,* and colistin lipid A binding in colistin-resistant *V. alginolyticus, E. coli, E. tarda, and K. pneumoniae* (55–57). Furthermore, metabolite supplementation can improve antibiotic uptake by enhancing porin expression; for example, glutamine and inosine increase β-lactam killing in *E. coli* by upregulating the OmpF porin, and inosine increases tetracycline killing of *K. pneumoniae* by upregulating the OmpK 36 porin (55, 58).

Interestingly, itaconate improves *P. aeruginosa* resistance toward aminoglycosides by an undescribed mechanism but can inhibit bacterial growth in other gram-negative and gram-positive bacteria, most of which are unable to catabolize itaconate (194–196). Itaconate inhibits the glyoxylate cycle enzyme isocitrate lyase in *E. coli* and *S*. Typhimurium and slows energy metabolism in *S. aureus*, which consequently induces aminoglycoside tolerance in *S. aureus* (194, 195, 197).

Certain metabolites can also promote ROS accumulation. Pyruvate potentiates aminoglycoside lethality against *E. tarda, P. aeruginosa, E. coli, K. pneumoniae,* and methicillin-resistant *S. aureus* by boosting the pyruvate-cysteine-GSH system/glycine-ROS metabolic pathway, which results in ROS generation (59). Nitrite activates the pyruvate cycle and electron transport chain, inducing ROS generation while repressing antioxidants in *P. aeruginosa*, potentiating cefoperazone-sulbactam against *P. aeruginosa* resistant to that combination (60). Together, these findings highlight that metabolites in high abundance in the bacterial environment can alter cell survival by altering antibiotic uptake and increasing ROS.

#### Potential strategies based on central carbon and nitrogen metabolism-linked metabolite-mediated alteration of antibiotic responses

Co-administering antibiotics with key metabolites to reprogram the metabolism of antibiotic-resistant, tolerant, or persister bacteria into susceptible states is a promising strategy to improve the therapeutic outcome of current antibiotics. Aminoglycosides combined with metabolite intermediates from the central carbon metabolism have reduced the bacterial load and improved host survival in mouse, zebrafish, and Huiyang bearded chicken infection models infected with *V. alginolyticus, Salmonella* spp.*,* or *E. tarda* (13, 47–51, 53). The combination of pyruvate with colistin to treat colistin-resistant *V. alginolyticus* in a zebrafish infection model yielded a higher survival rate when compared to the colistin-treated group (56). Ampicillin combined with fructose or glutamine improved the survival of zebrafish infected with ampicillin-resistant *S. agalactiae* and increased the survival of mice infected with *E. coli*, *P. aeruginosa*, or *K. pneumoniae* while also reducing the bacterial load in blood, liver, and spleen (57, 58). Inosine-tetracycline combination improved mouse survival by fivefold in a peritonitis-sepsis infection model and resulted in a lower bacterial load in the heart, lung, and liver when compared to tetracycline treatment (55). These data support the potential use of this strategy to combat antibiotic resistance and persister infections. Notably, the metabolites achieved *in vivo* effectiveness at concentrations well below previously reported tolerable amounts in mammals in some of the studies reported herein (47, 58, 198, 199), suggesting that this strategy could provide a safe and effective potential therapeutic application.

### Microbial secreted volatile-mediated communication of resistance

**Fig 5:**
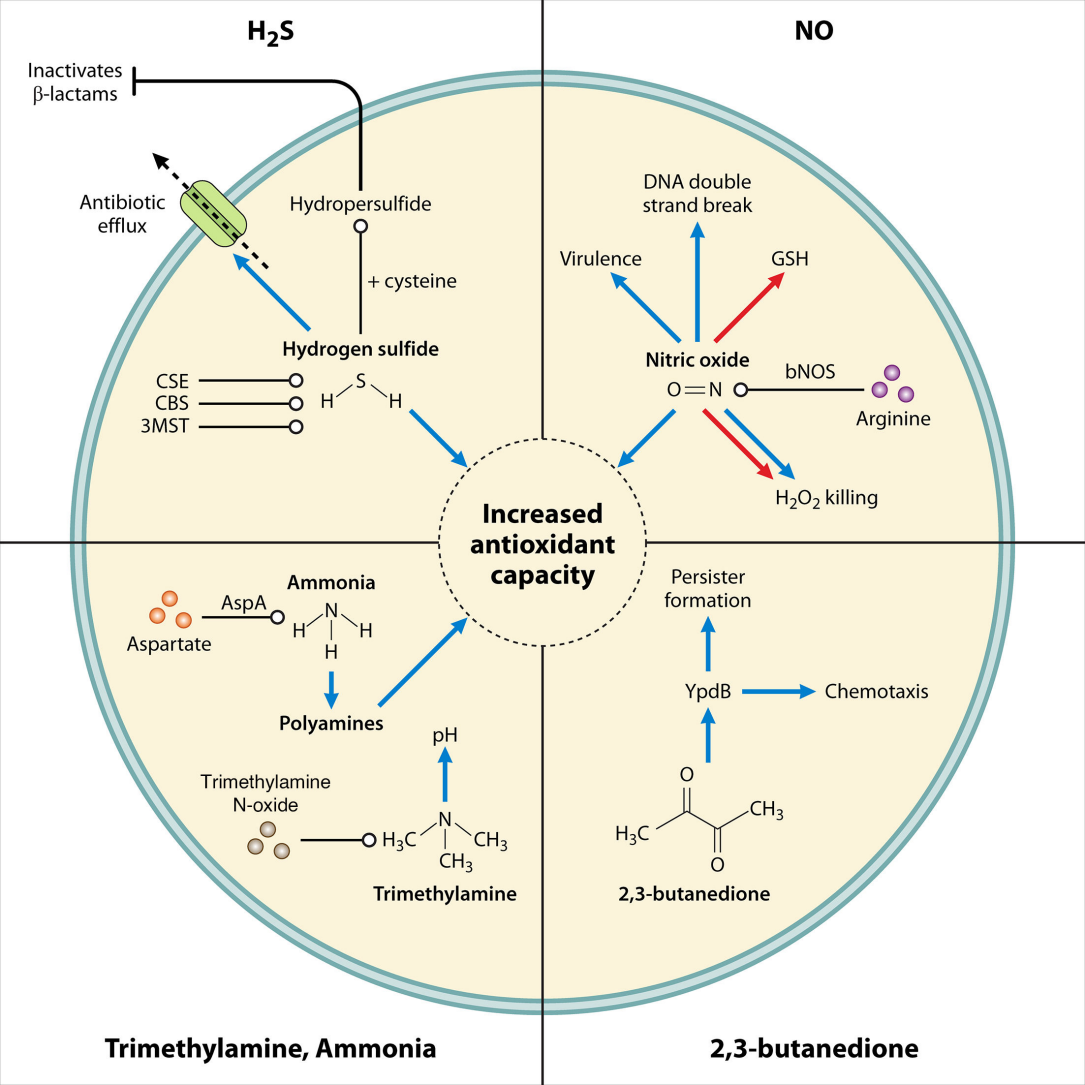
Mechanisms of VOC-mediated alteration of antibiotic susceptibility. H_2_S-mediated antibiotic resistance is attributed to H_2_S-mediated increase in antioxidant capacity and the oxidation of H_2_S into sulfane sulfur, which de-represses antibiotic efflux pumps. H_2_S can also react with cysteine to produce cysteine hydropersulfide, which degrades β-lactams into inactive forms. NO potentiated H_2_O_2_ killing by increasing double-strand DNA breaks and decreasing GSH concentrations. Conversely, NO can protect bacteria from H_2_O_2_ by increasing the antioxidant capacity of cells. It can also protect bacteria from β-lactams. The loss of bacterial nitric oxide synthase attenuates virulence in macrophages. Both ammonia and TMA protect gram-negative and gram-positive bacteria from tetracycline, whereby, in *E. coli*, ammonia promotes polyamine synthesis, increasing the antioxidant capacity of the cell, and TMA increases cytoplasmic pH, reducing tetracycline uptake. 2,3-butanedione induces persister formation in *E. coli* upon β-lactam and tetracycline exposure, potentially by regulating ypdB. For detailed descriptions of each mechanism and the corresponding references, refer to the text. Blue arrows denote increased, red arrows denote decreased, blunt-head arrows denote inhibited, dotted arrows denote the movement of a substrate, black flat-headed arrows denote inhibition, open circled arrows denote synthesis, and the blue membrane denotes both a gram-negative and gram-positive membrane.

In recent years, there has been an emerging interest in complex volatile compounds playing a role in bacterial interactions (200). Volatile compounds constitute a large class of infochemicals (chemicals involved in signaling, communication, and transmission of information between cells) characterized by their high vapor pressure, low boiling point, and low molar mass (201, 202). A database of identified microbial secreted VOCs has been published (201). This section will review VOCs relevant to human bacterial infection shown to alter antibiotic resistance, tolerance, or persistence, namely nitric oxide (NO), hydrogen sulfide (H_2_S), ammonia, 2,3-butanedione, and trimethylamine (TMA) (Fig. 5).

#### Hydrogen sulfide alteration of antibiotic responses

Loss of H_2_S production through chemical or genetic inactivation of cystathionine γ-lyase (CSE), cystathionine β-synthase (CBS), and 3-mercaptopyruvate sulfurtransferase (3-MST) resulted in increased *in vitro* susceptibility of the pathogens *Bacillus anthracis, P. aeruginosa, S. aureus,* and *E. coli* to aminoglycosides, β-lactams, fluoroquinolones, and H_2_O_2_, which was chemically complemented by exogenous H_2_S (61, 63, 203). Of note, genetic perturbation of H_2_S production in *P. aeruginosa* PAO1 by deletion or overexpression in another study yielded no change in antibiotic susceptibility (204). Chemical inactivation of bacterial CSE, CBS, and 3-MST was achieved by DL-propargylglycine (PAG), amino-oxyacetate (AOAA), and aspartate (Asp), respectively (61, 203). In contrast, Weikum and colleagues (205) reported that exogenous H_2_S synergized with tetracyclines, quinolones, and β-lactams against *S. aureus* in disk diffusion assays; they only observed sulfide-mediated protection from aminoglycosides, yet the inhibitor AOAA did not synergize with gentamicin in checkerboard assays, nor did gentamicin induce H_2_S production. Previously, PAG and AOAA were both combined with gentamicin to show synergy against *S. aureus* in bacterial killing experiments (203). The conflicting findings are likely due to the type of assays performed and strain variations, especially since *S. aureus* HG003, tested by Weikum et al. (205), did not exhibit substantial intracellular H_2_S levels. Interestingly, intracellular H_2_S production levels of several *E. coli* urinary tract infection clinical isolates positively correlated with their extents of antibiotic resistance (61). Conversely, H_2_S levels were lower in resistant and multidrug-resistant *P. aeruginosa* clinical isolates from cystic fibrosis patients relative to sensitive isolates (204). Together, the effects of H_2_S on antibiotic susceptibility may not be universal.

Various mechanisms contribute to H_2_S-mediated antibiotic resistance. Treatment with either macrolides or H_2_O_2_ induced H_2_S synthesis, and in turn, H_2_S stimulated the activities of catalase and superoxide dismutase and inhibited the Fenton reaction, which generates ROS (203). Additionally, H_2_S delayed killing of *E. coli* cells by antibiotics in bacterial *in vitro* time-kill analyses by downregulating cytochrome bo oxidase and inducing cytochrome bd oxidase I/II to maintain respiratory flux and redox balance and bolster the bacterial antioxidant capacity (61). The oxidation of H_2_S produces sulfane sulfur, which has been shown to react with regulatory thiols of the transcriptional regulator MexR in *P. aeruginosa,* leading to de-repression of MexAB-OprM efflux pumps, which increases antibiotic resistance (206). H_2_S can also react with cysteine to produce cysteine hydropersulfide, which can decompose β-lactams into their inactive form, contributing to intrinsic bacterial resistance to β-lactams (207).

#### Nitric oxide alteration of antibiotic responses

Nitric oxide is a soluble gas that is produced at the infection site by both the host macrophage cytokine-inducible nitric oxide synthase (iNOS) and the infecting microbe bacterial nitric oxide synthase (bNOS) (208, 209). Macrophages and neutrophils kill ingested pathogens using a multitude of factors, which include H_2_O_2_ oxidative stress (208). During infection, excess NO is produced by iNOS; iNOS-deficient mice display increased bacterial growth and susceptibility to infection compared to wild-type mice, suggesting NO has a role in host innate resistance to infection (210).

NO both synergized and antagonized H_2_O_2_ against different bacteria. Exogenous NO has been shown to potentiate H_2_O_2_ killing of *E. coli* (211–213). *E. coli* treated with the NO donor diethylamine/NO exhibited no cytotoxicity, but the addition of H_2_O_2_ led to a 10,000-fold increase in bactericidal effects (211). The synergistic combination of NO and H_2_O_2_ led to increased double-strand DNA breaks, altered cellular respiration, and decreased antioxidant GSH concentration, and indirect evidence suggested metal ions played a key role in the NO/H_2_O_2_ bactericidal mechanism (211, 213). Conversely, 30 µM NO increased the resistance of *B. subtilis* to 10 mM H_2_O_2_ (lethal dose) by 100-fold (214). The protection was hypothesized to be through a dual mechanism, whereby NO boosts the activity of pre-existing H_2_O_2_ scavenging enzymes and inhibits the Fenton reaction by transiently interrupting cysteine reduction by inhibiting Trx/TrxRed (214). Interestingly, this mechanism is similar to that of H_2_S, and NO-deficient *B. anthracis* Δ*nos* cells had increased H_2_S production, suggesting that both systems share a role in bacterial response to ROS (203). Moreover, *B. anthracis* uses bNOS-generated NO to combat host immune oxidative stress, and the loss of bNOS greatly attenuates virulence (215).

Several bacteria produce NO endogenously via bNOS (216). *B. subtilis* Δ*bNOS* exhibits reduced bacterial growth compared to the wild type when challenged with β-lactam, aminoglycoside, and quinolone antibiotics, and exogenous 100 µM NO temporarily protects the cells from β-lactam toxicity (217). *S. aureus* Δ*bNOS* was also more susceptible to β-lactams in turbidimetric and CFU counting time-dependent assays (217). It was also shown indirectly that treatment of β-lactams in *B. subtilis* stimulates bNOS activity (217). The mechanisms of NO-mediated alteration of antibiotic susceptibility are yet to be fully explored.

#### Ammonia and TMA alteration of antibiotic responses

Placing *E. coli* spent media in one compartment next to bacteria cultured in another compartment in two-petri-dish assays led to protection of *E. coli*, *P. aeruginosa*, *B. subtilis*, and *S. aureus* from tetracycline due to aerial exposure to volatile compounds produced by *E. coli* (110, 218). Testing pure volatile compounds known to be released from *E. coli* revealed that ammonia and TMA both induced tetracycline resistance (110, 218). Spent media of *E. coli* with disrupted ammonia production (Δ*aspC*, Δ*purA*, or Δ*aspA*) did not aerially induce tetracycline resistance (110). Aerial ammonia increased the intracellular pool of polyamines, and impairment of polyamine biosynthesis significantly reduced the protective effects of aerial ammonia (110). In addition, ammonia could combat oxidative stress induced by paraquat, similar to polyamines (110). Taken together, ammonia-induced tetracycline resistance appears to be a result of polyamine-induced protection (110).

To show the effects of TMA independent of aerial ammonia, an *aspC E. coli* mutant was used in the presence of TMA N-oxide (TMAO, a TMA substrate) as the VOC donor protected *E. coli*, *P. aeruginosa*, *B. subtilis*, and *S. aureus* from tetracycline and chloramphenicol; tetracycline resistance was lost upon disruption of TMAO to TMA conversion (218). The effects of TMA were not dependent on polyamine biosynthesis and did not reduce paraquat oxidative stress (218). The resulting tetracycline protection was shown to be a result of increased pH, causing a reduction in tetracycline uptake (218).

#### 2,3-butanedione alteration of antibiotic responses

The VOC 2,3-butanedione, emitted by *S. aureus*, *E. coli*, and *K. pneumoniae*, has been shown to induce *E. coli* persister cell formation upon treatment with β-lactam and tetracycline antibiotics (219–221). The dominant factor for 2,3-butanedione persister formation is the induction of *hipA* and *hipB* genes; *hipA* overexpression is a known mediator of persister formation (221). Furthermore, the exposure of *E. coli* to 2,3-butanedione downregulated all 30 genes associated with chemotaxis and motility (221). Interestingly, an *E. coli ypdB* knockout mutant exposed to 2,3-butanedione exhibited no change in swarming motility and persister cell formation, suggesting it may be a regulator of 2,3-butanedione effects in *E. coli* (221).

#### Potential targets from VOC alteration of antibiotic susceptibility

Leveraging VOC-related phenotypes may provide new potential antimicrobial strategies. Aspartate, a known inhibitor of 3-MST, suppressed the growth of *E. coli* in the presence of ampicillin (61). Inhibitors of 3-MST, CBS, and CSE enzymes showed enhanced *in vitro* clearance of *S. aureus* by leukocytes (62). CSE is the primary source of H_2_S production in *S. aureus* and *P. aeruginosa,* and CSE inhibitors in these pathogens have been shown to potentiate aminoglycosides in both *in vitro* and in a murine infection model (Table 1) (63). Additionally, the genetic or chemical inactivation of CSE lowers persister viability upon fluoroquinolone treatment (63). A double deletion of *cbs* and *cse* in *S. aureus* and deletion of the 3-MST homolog in *E. coli* (Δ*sseA*) exhibited potentiated host rapid immune-mediated killing and improved bacterial clearance in severe burn models (62). The drug delivery vector Gm@UiO-66-MA, which sequesters bacterially produced H_2_S, enhances the susceptibility of tolerant *E. coli* bacteria to aminoglycosides in *in vitro* and *in vivo* models (64). Additionally, a compound called 7B that scavenges H_2_S increases bacterial killing of *E. coli*, *S. aureus*, and *P. aeruginosa* when challenged with gentamicin and ciprofloxacin (65). 7B also marginally boosts bacterial clearance from macrophages and polymorphonuclear neutrophils and synergizes with gentamicin in a *P. aeruginosa*-infected pneumonia mouse model (65).

There are no bNOS inhibitors known to date, but NO-releasing polymeric implant devices potentiated various antibiotics against *S. aureus, P. aeruginosa*, *E. coli, B. subtilis, Enterococcus faecalis, Haemophilus influenzae*, and *A. baumannii* biofilms (reviewed extensively in references 66–68). On the other hand, PurA may serve as a target for inhibition of ammonia production; aurodox has been recently shown to inhibit PurA but exhibits insufficient target selectivity (69). PurA has also recently been recognized as a potential adjuvant drug target for colistin in *E. coli* (70). Furthermore, targeting YpdB, the potential master regulator of 2,3-butanedione effects on *E. coli,* may serve as a potential adjuvant target that has not yet been explored.

## LIMITATIONS, FUTURE DIRECTIONS, AND CONCLUDING REMARKS

Chemical-mediated alteration of antibiotic resistance is an emerging field in which discoveries may elucidate novel drug targets. AMR is an accelerating problem, compounded by the inherent finite number of druggable targets within a biological system; hence, exploring and exploiting all potential targets and therapeutic strategies is needed to combat infection. The concept of adjuvant compounds that inhibit bacterial resistance mechanisms provides an appealing strategy to revitalize current treatment regimens. Given the advances reviewed herein in our understanding of the role signaling compounds play in antibiotic resistance, tolerance, and persister formation, these small molecular cues offer a promising avenue for new drug targets.

Despite recent progress in this emerging field, several areas remain in need of clarification. For example, the field would benefit from clarification of the effective concentrations of these small molecular cues in the bacterial microenvironment at the infection site, which would better guide appropriate concentrations for *in vitro* assays. The concentration of these compounds may be body site-dependent, and a fraction of some chemicals, such as polyamines, may be bound to macromolecules, possibly complicating the determination of the free-molecule concentration. Nonetheless, *in vitro* phenotypes of some chemicals described in this review, tested at concentrations within their *in vivo* concentration ranges, have been validated in *in vivo* assays. Together, accurately identifying *in vivo* levels of the various small molecules and establishing *in vivo* models with adequate controls to validate *in vitro* phenotypes will improve clarity related to the effects of small molecules on antibiotic therapeutic outcomes.

Another limitation to our current understanding of chemically altered antibiotic susceptibility phenotypes is that they are mostly derived from lab-adapted reference strains with limited data from clinical isolates or diverse bacterial strains. As such, some strain-specific phenotypes may be erroneously generalized, and chemical-mediated antibiotic phenotypes and molecular mechanisms stemming from differences in regulatory components or other structures across bacteria may be overlooked. Therefore, it is advisable to validate the phenotypes of interest and their mechanisms across diverse sets of bacterial isolates.

Many of the potential targets described above require further validation and characterization; the inhibitors of such targets need testing to ensure *in vivo* efficacy and limited off-target effects. Additionally, the potential for compounding effects of multiple chemicals at the infection site, as previously shown with polyamines and bicarbonate (25), could be missed if the effects of one compound are minimal or dependent on the presence of another. Notably, the potential cumulative effects of chemicals at the site of infection on bacterial responses to antibiotics could range from synergistic (potentially leading to drastic antibiotic susceptibility shifts) to antagonistic (canceling the effects of one another). These possibilities warrant in-depth assessments of the combinatorial effects of host chemicals and others that bacteria encounter at the site of infection. The often complex and variable nature of phenotypes across bacterial type, growth stage, polymicrobial environment composition, and infection location can make targeting chemical-mediated effects challenging. It is plausible that inhibiting one form of chemical-mediated resistance, which is used as a chemical signal in one organism, could create a niche for another organism to take over.

Several additional questions remain in this area of research, including

- What other small molecules, secreted by bacteria or the host that bacteria encounter during infection, can modulate antibiotic resistance? What is the influence of exposure to such small molecules on antibiotic therapeutic outcomes *in vivo*?
- What are the exact molecular mechanisms by which the small molecules exert their effects on antibiotic resistance?
- How do bacteria sense and respond to these chemicals in the context of antibiotic susceptibility? What are the regulatory cascades that link the uptake, biosynthesis, efflux, and catabolism of these molecules to the antibiotic response?
- What are the combined effects of the different chemicals that might co-exist at the site of infection on bacterial response to antibiotics?
- Is there a hierarchy in the response of different bacteria to these signals that might influence the potential cumulative effects of chemicals at the site of infection and the development of a survival niche for specific organisms and not others?

Addressing these questions and clarifying the limitations outlined above will further enhance our understanding of the chemical-mediated alterations in antibiotic response and their potential influence on antibiotic therapeutic outcomes. Together, elucidating the mechanisms of chemical-mediated resistance in bacteria is a largely overlooked avenue that may offer new, much-needed therapeutic strategies to curb the rise of AMR and combat bacterial infections.

## References

[1] Public Health Agency of Canada. 2023. Pan-canadian action plan on antimicrobial resistance. Available from: https://www.canada.ca/en/public-health/services/publications/drugs-health-products/pan-canadian-action-plan-antimicrobial-resistance.html

[2] World Health Organization. 2019. Antibacterial agents in clinical development: an analysis of the antibacterial clinical development pipeline. Genrva World Health Organization

[3] Centers for Disease Control and Prevention. 2022. Antimicrobial resistance threats in the United States, 2021-2022. CDC, Atlanta, GA Department of Health and Human Services

[4] Naghavi M, Vollset SE, Ikuta KS, Swetschinski LR, Gray AP, Wool EE, Robles Aguilar G, Mestrovic T, Smith G, Han C, et al.. 2024. Global burden of bacterial antimicrobial resistance 1990–2021: a systematic analysis with forecasts to 2050. Lancet 404:1199–1226. doi:10.1016/S0140-6736(24)01867-1 39299261 PMC11718157

[5] Nikaido H. 2001. Preventing drug access to targets: cell surface permeability barriers and active efflux in bacteria. Semin Cell Dev Biol 12:215–223. doi:10.1006/scdb.2000.0247 11428914

[6] Dhanda G, Acharya Y, Haldar J. 2023. Antibiotic adjuvants: a versatile approach to combat antibiotic resistance. ACS Omega 8:10757–10783. doi:10.1021/acsomega.3c00312 37008128 PMC10061514

[7] Wright GD. 2016. Antibiotic adjuvants: rescuing antibiotics from resistance. Trends Microbiol 24:862–871. doi:10.1016/j.tim.2016.06.009 27430191

[8] El-Halfawy OM, Valvano MA. 2012. Non-genetic mechanisms communicating antibiotic resistance: rethinking strategies for antimicrobial drug design. Expert Opin Drug Discov 7:923–933. doi:10.1517/17460441.2012.712512 22860901

[9] Doern GV, Brecher SM. 2011. The clinical predictive value (or Lack Thereof) of the results of in vitro antimicrobial susceptibility tests. J Clin Microbiol 49:S11–S14. doi:10.1128/JCM.00580-11

[10] Papp-Wallace KM, Mack AR, Taracila MA, Bonomo RA. 2020. Resistance to novel β-lactam–β-lactamase inhibitor combinations: the “price of progress”. Infect Dis Clin North Am 34:773–819. doi:10.1016/j.idc.2020.05.001 33011051 PMC7609624

[11] El-Halfawy OM, Czarny TL, Flannagan RS, Day J, Bozelli JC, Kuiack RC, Salim A, Eckert P, Epand RM, McGavin MJ, Organ MG, Heinrichs DE, Brown ED. 2020. Discovery of an antivirulence compound that reverses β-lactam resistance in MRSA. Nat Chem Biol 16:143–149. doi:10.1038/s41589-019-0401-8 31768032

[12] Larsson DGJ, Flach C-F. 2022. Antibiotic resistance in the environment. Nat Rev Microbiol 20:257–269. doi:10.1038/s41579-021-00649-x 34737424 PMC8567979

[13] Peng B, Li H, Peng X-X. 2025. Metabolic state-driven nutrient-based approach to combat bacterial antibiotic resistance. NPJ Antimicrob Resist 3:24. doi:10.1038/s44259-025-00092-5 40185857 PMC11971349

[14] Chen X, Zhang L, Zhang M, Liu H, Lu P, Lin K. 2018. Quorum sensing inhibitors: a patent review (2014–2018). Expert Opin Ther Pat 28:849–865. doi:10.1080/13543776.2018.1541174 30366511

[15] Ó Muimhneacháin E, Reen FJ, O’Gara F, McGlacken GP. 2018. Analogues of Pseudomonas aeruginosa signalling molecules to tackle infections. Org Biomol Chem 16:169–179. doi:10.1039/c7ob02395b 29095463

[16] Sikdar R, Elias MH. 2022. Evidence for complex interplay between quorum sensing and antibiotic resistance in Pseudomonas aeruginosa. Microbiol Spectr 10:e0126922. doi:10.1128/spectrum.01269-22 36314960 PMC9769976

[17] Soukarieh F, Gurnani P, Romero M, Halliday N, Stocks M, Alexander C, Cámara M. 2023. Design of quorum sensing inhibitor-polymer conjugates to penetrate Pseudomonas aeruginosa biofilms. ACS Macro Lett 12:314–319. doi:10.1021/acsmacrolett.2c00699 36790191 PMC10035027

[18] Wang Y, Ma S. 2014. Small molecules modulating AHL-based quorum sensing to attenuate bacteria virulence and biofilms as promising antimicrobial drugs. Curr Med Chem 21:296–311. doi:10.2174/09298673113206660294 24164200

[19] El-Halfawy OM, Valvano MA. 2014. Putrescine reduces antibiotic-induced oxidative stress as a mechanism of modulation of antibiotic resistance in Burkholderia cenocepacia. Antimicrob Agents Chemother 58:4162–4171. doi:10.1128/AAC.02649-14 24820075 PMC4068564

[20] Bacchi CJ, Nathan HC, Hutner SH, McCann PP, Sjoerdsma A. 1980. Polyamine metabolism: a potential therapeutic target in trypanosomes. Science 210:332–334. doi:10.1126/science.6775372 6775372

[21] Smego RA Jr, Nagar S, Maloba B, Popara M. 2001. A meta-analysis of salvage therapy for Pneumocystis carinii pneumonia. Arch Intern Med 161:1529–1533. doi:10.1001/archinte.161.12.1529 11427101

[22] Mayers JR, Varon J, Zhou RR, Daniel-Ivad M, Beaulieu C, Bhosle A, Glasser NR, Lichtenauer FM, Ng J, Vera MP, Huttenhower C, Perrella MA, Clish CB, Zhao SD, Baron RM, Balskus EP. 2024. A metabolomics pipeline highlights microbial metabolism in bloodstream infections. Cell 187:4095–4112. doi:10.1016/j.cell.2024.05.035 38885650 PMC11283678

[23] Moulding PB, Flannagan RS, Wong J, Soliman AM, Elhenawy W, Heinrichs DE, El-Halfawy OM. 2024. Discovery of broad-spectrum bacterial polyamine detoxification inhibitors as potential antivirulence agents and antibiotic adjuvants. bioRxiv. doi:10.1101/2024.10.18.618978

[24] Liao C-P, Phanstiel O IV, Lasbury ME, Zhang C, Shao S, Durant PJ, Cheng B-H, Lee C-H. 2009. Polyamine transport as a target for treatment of Pneumocystis pneumonia. Antimicrob Agents Chemother 53:5259–5264. doi:10.1128/AAC.00662-09 19805570 PMC2786323

[25] Adams JME, Moulding PB, El-Halfawy OM. 2024. Polyamine-mediated sensitization of Klebsiella pneumoniae to macrolides through a dual mode of action. ACS Infect Dis 10:2183–2195. doi:10.1021/acsinfecdis.4c00157 38695481

[26] Sabir S, Das T, Kuppusamy R, Yu TT, Willcox MD, Black DS, Kumar N. 2023. Novel quinazolinone disulfide analogues as pqs quorum sensing inhibitors against Pseudomonas aeruginosa. Bioorg Chem 130:106226. doi:10.1016/j.bioorg.2022.106226 36332317

[27] Murray EJ, Dubern J-F, Chan WC, Chhabra SR, Williams P. 2022. A Pseudomonas aeruginosa PQS quorum-sensing system inhibitor with anti-staphylococcal activity sensitizes polymicrobial biofilms to tobramycin. Cell Chem Biol 29:1187–1199. doi:10.1016/j.chembiol.2022.02.007 35259345 PMC9605878

[28] Liu J, Hou J-S, Chang Y-Q, Peng L-J, Zhang X-Y, Miao Z-Y, Sun P-H, Lin J, Chen W-M. 2022. New Pqs quorum sensing system inhibitor as an antibacterial synergist against multidrug-resistant Pseudomonas aeruginosa. J Med Chem 65:688–709. doi:10.1021/acs.jmedchem.1c01781 34951310

[29] Hamed MM, Abdelsamie AS, Rox K, Schütz C, Kany AM, Röhrig T, Schmelz S, Blankenfeldt W, Arce-Rodriguez A, Borrero-de Acuña JM, Jahn D, Rademacher J, Ringshausen FC, Cramer N, Tümmler B, Hirsch AKH, Hartmann RW, Empting M. 2023. Towards translation of PqsR inverse agonists: from in vitro efficacy optimization to in vivo proof-of-principle. Adv Sci (Weinh) 10:e2204443. doi:10.1002/advs.202204443 36596691 PMC9929129

[30] Schütz C, Ho D-K, Hamed MM, Abdelsamie AS, Röhrig T, Herr C, Kany AM, Rox K, Schmelz S, Siebenbürger L, et al.. 2021. A new PqsR inverse agonist potentiates tobramycin efficacy to eradicate Pseudomonas aeruginosa biofilms. Adv Sci (Weinh) 8:e2004369. doi:10.1002/advs.202004369 34165899 PMC8224453

[31] Soukarieh F, Mashabi A, Richardson W, Oton EV, Romero M, Dubern J-F, Robertson SN, Lucanto S, Markham-Lee Z, Sou T, Kukavica-Ibrulj I, Levesque RC, Bergstrom CAS, Halliday N, Kellam B, Emsley J, Heeb S, Williams P, Stocks MJ, Cámara M. 2024. Design, synthesis, and evaluation of new 1H-benzo[d]Imidazole based PqsR inhibitors as adjuvant therapy for Pseudomonas aeruginosa infections. J Med Chem 67:1008–1023. doi:10.1021/acs.jmedchem.3c00973 38170170 PMC10823468

[32] Wen F, Wu Y, Yuan Y, Yang X, Ran Q, Gan X, Guo Y, Wang X, Chu Y, Zhao K. 2024. Discovery of psoralen as a quorum sensing inhibitor suppresses Pseudomonas aeruginosa virulence. Appl Microbiol Biotechnol 108:222. doi:10.1007/s00253-024-13067-9 38372782 PMC10876730

[33] Huang XH, She MT, Zhang YH, Liu YF, Zhong DX, Zhang YH, Zheng JX, Sun N, Wong WL, Lu YJ. 2022. Novel quinoline‐based derivatives as the PqsR inhibitor against Pseudomonas aeruginosa PAO1. J Appl Microbiol 133:2167–2181. doi:10.1111/jam.15601 35490292

[34] Maura D, Drees SL, Bandyopadhaya A, Kitao T, Negri M, Starkey M, Lesic B, Milot S, Déziel E, Zahler R, Pucci M, Felici A, Fetzner S, Lépine F, Rahme LG. 2017. Polypharmacology approaches against the Pseudomonas aeruginosa MvfR regulon and their application in blocking virulence and antibiotic tolerance. ACS Chem Biol 12:1435–1443. doi:10.1021/acschembio.6b01139 28379691 PMC12908516

[35] Thomann A, de Mello Martins AGG, Brengel C, Empting M, Hartmann RW. 2016. Application of dual inhibition concept within looped autoregulatory systems toward antivirulence agents against Pseudomonas aeruginosa infections. ACS Chem Biol 11:1279–1286. doi:10.1021/acschembio.6b00117 26882081

[36] Chen J, Lu Y, Ye F, Zhang H, Zhou Y, Li J, Wu Q, Xu X, Wu Q, Wei B, Zhang H, Wang H. 2022. A small-molecule inhibitor of the anthranilyl-CoA synthetase PqsA for the treatment of multidrug-resistant Pseudomonas aeruginosa. Microbiol Spectr 10:e02764-21. doi:10.1128/spectrum.02764-21 35856709 PMC9430567

[37] Dehbashi S, Tahmasebi H, Alikhani MY, Vidal JE, Seifalian A, Arabestani MR. 2024. The healing effect of Pseudomonas Quinolone Signal (PQS) with co-infection of Staphylococcus aureus and Pseudomonas aeruginosa: a preclinical animal co-infection model. J Infect Public Health 17:329–338. doi:10.1016/j.jiph.2023.12.016 38194764

[38] Law RJ, Hamlin JNR, Sivro A, McCorrister SJ, Cardama GA, Cardona ST. 2008. A functional phenylacetic acid catabolic pathway is required for full pathogenicity of Burkholderia cenocepacia in the Caenorhabditis elegans host model. J Bacteriol 190:7209–7218. doi:10.1128/JB.00481-08 18776009 PMC2580687

[39] Cerqueira GM, Kostoulias X, Khoo C, Aibinu I, Qu Y, Traven A, Peleg AY. 2014. A global virulence regulator in Acinetobacter baumannii and its control of the phenylacetic acid catabolic pathway. J Infect Dis 210:46–55. doi:10.1093/infdis/jiu024 24431277

[40] Hunt TA, Kooi C, Sokol PA, Valvano MA. 2004. Identification of Burkholderia cenocepacia genes required for bacterial survival in vivo. Infect Immun 72:4010–4022. doi:10.1128/IAI.72.7.4010-4022.2004 15213146 PMC427415

[41] Hooppaw AJ, McGuffey JC, Di Venanzio G, Ortiz-Marquez JC, Weber BS, Lightly TJ, van Opijnen T, Scott NE, Cardona ST, Feldman MF. 2022. The phenylacetic acid catabolic pathway regulates antibiotic and oxidative stress responses in Acinetobacter. mBio 13:e0186321. doi:10.1128/mbio.01863-21 35467424 PMC9239106

[42] Vega NM, Allison KR, Samuels AN, Klempner MS, Collins JJ. 2013. Salmonella typhimurium intercepts Escherichia coli signaling to enhance antibiotic tolerance. Proc Natl Acad Sci USA 110:14420–14425. doi:10.1073/pnas.1308085110 23946425 PMC3761632

[43] Graboski AL, Kowalewski ME, Simpson JB, Cao X, Ha M, Zhang J, Walton WG, Flaherty DP, Redinbo MR. 2023. Mechanism-based inhibition of gut microbial tryptophanases reduces serum indoxyl sulfate. Cell Chem Biol 30:1402–1413. doi:10.1016/j.chembiol.2023.07.015 37633277 PMC10702206

[44] Scherzer R, Gdalevsky GY, Goldgur Y, Cohen-Luria R, Bittner S, Parola AH. 2009. New tryptophanase inhibitors: towards prevention of bacterial biofilm formation. J Enzyme Inhib Med Chem 24:350–355. doi:10.1080/14756360802187612 18608755

[45] Do QT, Nguyen GT, Celis V, Phillips RS. 2014. Inhibition of Escherichia coli tryptophan indole-lyase by tryptophan homologues. Arch Biochem Biophys 560:20–26. doi:10.1016/j.abb.2014.07.027 25088962

[46] Allison KR, Brynildsen MP, Collins JJ. 2011. Metabolite-enabled eradication of bacterial persisters by aminoglycosides. Nature 473:216–220. doi:10.1038/nature10069 21562562 PMC3145328

[47] Peng B, Su Y, Li H, Han Y, Guo C, Tian Y, Peng X. 2015. Exogenous alanine and/or glucose plus kanamycin kills antibiotic-resistant bacteria. Cell Metab 21:249–262. doi:10.1016/j.cmet.2015.01.008 25651179

[48] Yong Y, Zhou Y, Liu K, Liu G, Wu L, Fang B. 2021. Exogenous citrulline and glutamine contribute to reverse the resistance of Salmonella to apramycin. Front Microbiol 12:2021. doi:10.3389/fmicb.2021.759170 PMC8552007 34721368

[49] Kuang S, Xiang J, Chen Y, Peng X, Li H, Peng B. 2024. Exogenous pyruvate promotes gentamicin uptake to kill antibiotic-resistant Vibrio alginolyticus. Int J Antimicrob Agents 63:107036. doi:10.1016/j.ijantimicag.2023.107036 37981076

[50] Zhang S, Wang J, Jiang M, Xu D, Peng B, Peng X-X, Li H. 2019. Reduced redox-dependent mechanism and glucose-mediated reversal in gentamicin-resistant Vibrio alginolyticus. Environ Microbiol 21:4724–4739. doi:10.1111/1462-2920.14811 31595636

[51] Su Y, Peng B, Han Y, Li H, Peng X. 2015. Fructose restores susceptibility of multidrug-resistant Edwardsiella tarda to kanamycin. J Proteome Res 14:1612–1620. doi:10.1021/pr501285f 25675328

[52] Zhang S, Yang M-J, Peng B, Peng X-X, Li H. 2020. Reduced ROS-mediated antibiotic resistance and its reverting by glucose in Vibrio alginolyticus. Environ Microbiol 22:4367–4380. doi:10.1111/1462-2920.15085 32441046

[53] Jiang M, Li X, Xie C-L, Chen P, Luo W, Lin C-X, Wang Q, Shu D-M, Luo C-L, Qu H, Ji J. 2023. Fructose-enabled killing of antibiotic-resistant Salmonella enteritidis by gentamicin: Insight from reprogramming metabolomics. Int J Antimicrob Agents 62:106907. doi:10.1016/j.ijantimicag.2023.106907 37385564

[54] Tang X-K, Su Y-B, Ye H-Q, Dai Z-Y, Yi H, Yang K-X, Zhang T-T, Chen Z-G. 2021. Glucose-potentiated amikacin killing of cefoperazone/sulbactam resistant Pseudomonas aeruginosa. Front Microbiol 12:800442. doi:10.3389/fmicb.2021.800442 35310395 PMC8928219

[55] Li F, Xu T, Fang D, Wang Z, Liu Y. 2024. Inosine reverses multidrug resistance in Gram-negative bacteria carrying mobilized RND-type efflux pump gene cluster tmexCD-toprJ. mSystems 9:e0079724. doi:10.1128/msystems.00797-24 39254032 PMC11495011

[56] Li L, Su Y-B, Peng B, Peng X-X, Li H. 2020. Metabolic mechanism of colistin resistance and its reverting in Vibrio alginolyticus. Environ Microbiol 22:4295–4313. doi:10.1111/1462-2920.15021 32291842

[57] Chen X-W, Wu J-H, Liu Y-L, Munang’andu HM, Peng B. 2023. Fructose promotes ampicillin killing of antibiotic-resistant Streptococcus agalactiae. Virulence 14:2180938. doi:10.1080/21505594.2023.2180938 36803528 PMC9980678

[58] Zhao X, Chen Z, Yang T, Jiang M, Wang J, Cheng Z, Yang M, Zhu J, Zhang T, Li H, Peng B, Peng X. 2021. Glutamine promotes antibiotic uptake to kill multidrug-resistant uropathogenic bacteria. Sci Transl Med 13:eabj0716. doi:10.1126/scitranslmed.abj0716 34936385

[59] Xiang J, Tian S, Wang S, Liu Y, Li H, Peng B. 2024. Pyruvate abundance confounds aminoglycoside killing of multidrug-resistant bacteria via glutathione metabolism. Research (Lambertville) 7:0554. doi:10.34133/research.0554 PMC11654824 39697188

[60] Kuang S-F, Li X, Feng D-Y, Wu W-B, Li H, Peng B, Peng X-X, Chen Z-G, Zhang T-T. 2022. Nitrite promotes ROS production to potentiate cefoperazone-sulbactam-mediated elimination to lab-evolved and clinical-evolved Pseudomonas aeruginosa. Microbiol Spectr 10:e0232721. doi:10.1128/spectrum.02327-21 35863024 PMC9430864

[61] Shukla P, Khodade VS, SharathChandra M, Chauhan P, Mishra S, Siddaramappa S, Pradeep BE, Singh A, Chakrapani H. 2017. “On demand” redox buffering by H_2_S contributes to antibiotic resistance revealed by a bacteria-specific H_2_S donor. Chem Sci 8:4967–4972. doi:10.1039/c7sc00873b 28959420 PMC5607856

[62] Toliver-Kinsky T, Cui W, Törö G, Lee SJ, Shatalin K, Nudler E, Szabo C. 2019. H_2_S, a bacterial defense mechanism against the host immune response. Infect Immun 87:e00272-18. doi:10.1128/IAI.00272-18 30323021 PMC6300618

[63] Shatalin K, Nuthanakanti A, Kaushik A, Shishov D, Peselis A, Shamovsky I, Pani B, Lechpammer M, Vasilyev N, Shatalina E, Rebatchouk D, Mironov A, Fedichev P, Serganov A, Nudler E. 2021. Inhibitors of bacterial H_2_S biogenesis targeting antibiotic resistance and tolerance. Science 372:1169–1175. doi:10.1126/science.abd8377 34112687 PMC10723041

[64] Huo S, Xie Q, Zhang M, Jiang Z, Fu L, Li W, Bian C, Wu K, Zhu Y, Nie X, Ding S. 2023. H_2_S-removing UiO-66 MOFs for sensitized antibacterial therapy. J Mater Chem B 11:5817–5829. doi:10.1039/D3TB00552F 37278619

[65] Sun J, Wang X, Gao Y, Li S, Hu Z, Huang Y, Fan B, Wang X, Liu M, Qiao C, Zhang W, Wang Y, Ji X. 2024. H_2_S scavenger as a broad-spectrum strategy to deplete bacteria-derived H_2_S for antibacterial sensitization. Nat Commun 15:9422. doi:10.1038/s41467-024-53764-7 39482291 PMC11527999

[66] Wang TY, Zhu XY, Wu FG. 2023. Antibacterial gas therapy: strategies, advances, and prospects. Bioact Mater 23:129–155. doi:10.1016/j.bioactmat.2022.10.008 36406249 PMC9661653

[67] Sadrearhami Z, Nguyen T-K, Namivandi-Zangeneh R, Jung K, Wong EHH, Boyer C. 2018. Recent advances in nitric oxide delivery for antimicrobial applications using polymer-based systems. J Mater Chem B 6:2945–2959. doi:10.1039/c8tb00299a 32254331

[68] Chug MK, Brisbois EJ. 2022. Recent developments in multifunctional antimicrobial surfaces and applications toward advanced nitric oxide-based biomaterials. ACS Mater Au 2:525–551. doi:10.1021/acsmaterialsau.2c00040 36124001 PMC9479141

[69] Watanabe Y, Haneda T, Kimishima A, Kuwae A, Suga T, Suzuki T, Iwabuchi Y, Honsho M, Honma S, Iwatsuki M, Matsui H, Hanaki H, Kanoh N, Abe A, Asami Y, Ōmura S. 2024. PurA is the main target of aurodox, a type III secretion system inhibitor. Proc Natl Acad Sci USA 121:e2322363121. doi:10.1073/pnas.2322363121 38640341 PMC11046696

[70] Kano T, Ishikawa K, Furuta K, Kaito C. 2024. Knockout of adenylosuccinate synthase purA increases susceptibility to colistin in Escherichia coli. FEMS Microbiol Lett 371:fnae007. doi:10.1093/femsle/fnae007 38305138 PMC10876104

[71] Feuerstein BG, Williams LD, Basu HS, Marton LJ. 1991. Implications and concepts of polyamine-nucleic acid interactions. J Cell Biochem 46:37–47. doi:10.1002/jcb.240460107 1874798

[72] Wortham BW, Patel CN, Oliveira MA. 2007. Polyamines in bacteria: pleiotropic effects yet specific mechanisms. Adv Exp Med Biol 603:106–115. doi:10.1007/978-0-387-72124-8_9 17966408

[73] Zhang M, Wang H, Tracey KJ. 2000. Regulation of macrophage activation and inflammation by spermine: a new chapter in an old story. Crit Care Med 28:N60–N66. doi:10.1097/00003246-200004001-00007 10807317

[74] Eisenberg T, Knauer H, Schauer A, Büttner S, Ruckenstuhl C, Carmona-Gutierrez D, Ring J, Schroeder S, Magnes C, Antonacci L, et al.. 2009. Induction of autophagy by spermidine promotes longevity. Nat Cell Biol 11:1305–1314. doi:10.1038/ncb1975 19801973

[75] Thurlow LR, Joshi GS, Clark JR, Spontak JS, Neely CJ, Maile R, Richardson AR. 2013. Functional modularity of the arginine catabolic mobile element contributes to the success of USA300 methicillin-resistant Staphylococcus aureus. Cell Host Microbe 13:100–107. doi:10.1016/j.chom.2012.11.012 23332159 PMC3553549

[76] Watanabe S, Kusama-Eguchi K, Kobayashi H, Igarashi K. 1991. Estimation of polyamine binding to macromolecules and ATP in bovine lymphocytes and rat liver. J Biol Chem 266:20803–20809. doi:10.1016/S0021-9258(18)54780-3 1718969

[77] Igarashi K, Kashiwagi K. 2010. Modulation of cellular function by polyamines. Int J Biochem Cell Biol 42:39–51. doi:10.1016/j.biocel.2009.07.009 19643201

[78] Hasan CM, Pottenger S, Green AE, Cox AA, White JS, Jones T, Winstanley C, Kadioglu A, Wright MH, Neill DR, Fothergill JL. 2022. Pseudomonas aeruginosa utilizes the host-derived polyamine spermidine to facilitate antimicrobial tolerance. JCI Insight 7:e158879. doi:10.1172/jci.insight.158879 36194492 PMC9746822

[79] Dwyer DJ, Belenky PA, Yang JH, MacDonald IC, Martell JD, Takahashi N, Chan CTY, Lobritz MA, Braff D, Schwarz EG, Ye JD, Pati M, Vercruysse M, Ralifo PS, Allison KR, Khalil AS, Ting AY, Walker GC, Collins JJ. 2014. Antibiotics induce redox-related physiological alterations as part of their lethality. Proc Natl Acad Sci USA 111:E2100–E2109. doi:10.1073/pnas.1401876111 24803433 PMC4034191

[80] Akhova A, Nesterova L, Shumkov M, Tkachenko A. 2021. Cadaverine biosynthesis contributes to decreased Escherichia coli susceptibility to antibiotics. Res Microbiol 172:103881. doi:10.1016/j.resmic.2021.103881 34543694

[81] Tkachenko AG, Akhova AV, Shumkov MS, Nesterova LY. 2012. Polyamines reduce oxidative stress in Escherichia coli cells exposed to bactericidal antibiotics. Res Microbiol 163:83–91. doi:10.1016/j.resmic.2011.10.009 22138596

[82] Solmi L, Rossi FR, Romero FM, Bach-Pages M, Preston GM, Ruiz OA, Gárriz A. 2023. Polyamine-mediated mechanisms contribute to oxidative stress tolerance in Pseudomonas syringae. Sci Rep 13:4279. doi:10.1038/s41598-023-31239-x 36922543 PMC10017717

[83] Chang W, Small DA, Toghrol F, Bentley WE. 2005. Microarray analysis of Pseudomonas aeruginosa reveals induction of pyocin genes in response to hydrogen peroxide. BMC Genomics 6:115. doi:10.1186/1471-2164-6-115 16150148 PMC1250226

[84] Tkachenko A, Nesterova L, Pshenichnov M. 2001. The role of the natural polyamine putrescine in defense against oxidative stress in Escherichia coli. Arch Microbiol 176:155–157. doi:10.1007/s002030100301 11479716

[85] Chattopadhyay MK, Tabor CW, Tabor H. 2003. Polyamines protect Escherichia coli cells from the toxic effect of oxygen. Proc Natl Acad Sci USA 100:2261–2265. doi:10.1073/pnas.2627990100 12591940 PMC151328

[86] Jung IL, Kim IG. 2003. Transcription of ahpC, katG, and katE genes in Escherichia coli is regulated by polyamines: polyamine-deficient mutant sensitive to H_2_O_2_-induced oxidative damage. Biochem Biophys Res Commun 301:915–922. doi:10.1016/s0006-291x(03)00064-0 12589799

[87] Sakamoto A, Terui Y, Yoshida T, Yamamoto T, Suzuki H, Yamamoto K, Ishihama A, Igarashi K, Kashiwagi K. 2015. Three members of polyamine modulon under oxidative stress conditions: two transcription factors (SoxR and EmrR) and a glutathione synthetic enzyme (GshA). PLoS One 10:e0124883. doi:10.1371/journal.pone.0124883 25898225 PMC4405209

[88] Minton KW, Tabor H, Tabor CW. 1990. Paraquat toxicity is increased in Escherichia coli defective in the synthesis of polyamines. Proc Natl Acad Sci USA 87:2851–2855. doi:10.1073/pnas.87.7.2851 2181453 PMC53789

[89] Johnson L, Mulcahy H, Kanevets U, Shi Y, Lewenza S. 2012. Surface-localized spermidine protects the Pseudomonas aeruginosa outer membrane from antibiotic treatment and oxidative stress. J Bacteriol 194:813–826. doi:10.1128/JB.05230-11 22155771 PMC3272965

[90] Barbagallo M, Di Martino ML, Marcocci L, Pietrangeli P, De Carolis E, Casalino M, Colonna B, Prosseda G. 2011. A new piece of the Shigella pathogenicity puzzle: spermidine Accumulationby silencing of the speG gene. PLoS One 6:e27226. doi:10.1371/journal.pone.0027226 22102881 PMC3213128

[91] Ha HC, Sirisoma NS, Kuppusamy P, Zweier JL, Woster PM, Casero RA Jr. 1998. The natural polyamine spermine functions directly as a free radical scavenger. Proc Natl Acad Sci USA 95:11140–11145. doi:10.1073/pnas.95.19.11140 9736703 PMC21609

[92] Das KC, Misra HP. 2004. Hydroxyl radical scavenging and singlet oxygen quenching properties of polyamines. Mol Cell Biochem 262:127–133. doi:10.1023/b:mcbi.0000038227.91813.79 15532717

[93] Drolet G, Dumbroff EB, Legge RL, Thompson JE. 1986. Radical scavenging properties of polyamines. Phytochemistry 25:367–371. doi:10.1016/S0031-9422(00)85482-5

[94] Tkachenko AG, Nesterova LY. 2003. Polyamines as modulators of gene expression under oxidative stress in Escherichia coli. Biochemistry (Mosc) 68:850–856. doi:10.1023/a:1025790729797 12948384

[95] Tkachenko AG, Kashevarova NM, Tyuleneva EA, Shumkov MS. 2017. Stationary-phase genes upregulated by polyamines are responsible for the formation of Escherichia coli persister cells tolerant to netilmicin. FEMS Microbiol Lett 364:fnx084. doi:10.1093/femsle/fnx084 28431088 PMC5827576

[96] Tkachenko AG. 2004. Mechanisms of protective functions of Escherichia coli polyamines against toxic effect of paraquat, which causes superoxide stress. Biochemistry (Moscow) 69:188–194. doi:10.1023/B:BIRY.0000018950.30452.53 15000686

[97] Tkachenko AG, Kashevarova NM, Karavaeva EA, Shumkov MS. 2014. Putrescine controls the formation of Escherichia coli persister cells tolerant to aminoglycoside netilmicin. FEMS Microbiol Lett 361:25–33. doi:10.1111/1574-6968.12613 25283595

[98] Seixas AF, Quendera AP, Sousa JP, Silva AFQ, Arraiano CM, Andrade JM. 2021. Bacterial response to oxidative stress and RNA oxidation. Front Genet 12:821535. doi:10.3389/fgene.2021.821535 35082839 PMC8784731

[99] Yao X, Li C, Zhang J, Lu C-D. 2012. γ-glutamyl spermine synthetase PauA2 as a potential target of antibiotic development against Pseudomonas aeruginosa. Antimicrob Agents Chemother 56:5309–5314. doi:10.1128/AAC.01158-12 22869561 PMC3457366

[100] Kumar V, Mishra RK, Ghose D, Kalita A, Dhiman P, Prakash A, Thakur N, Mitra G, Chaudhari VD, Arora A, Dutta D. 2022. Free spermidine evokes superoxide radicals that manifest toxicity. eLife 11:e77704. doi:10.7554/eLife.77704 35416771 PMC9038194

[101] Kwon DH, Hekmaty S, Seecoomar G. 2013. Homeostasis of glutathione is associated with polyamine-mediated β-lactam susceptibility in Acinetobacter baumannii ATCC 19606. Antimicrob Agents Chemother 57:5457–5461. doi:10.1128/AAC.00692-13 23979736 PMC3811305

[102] Malone L, Kwon DH. 2013. Carbapenem-associated multidrug-resistant Acinetobacter baumannii are sensitised by aztreonam in combination with polyamines. Int J Antimicrob Agents 41:70–74. doi:10.1016/j.ijantimicag.2012.08.009 23148986 PMC3530022

[103] Tkachenko AG, Pozhidaeva ON, Shumkov MS. 2006. Role of polyamines in formation of multiple antibiotic resistance of Escherichia coli under stress conditions. Biochemistry (Moscow) 71:1042–1049. doi:10.1134/S0006297906090148 17009960

[104] Kwon DH, Lu CD. 2007. Polyamine effects on antibiotic susceptibility in bacteria. Antimicrob Agents Chemother 51:2070–2077. doi:10.1128/AAC.01472-06 17438056 PMC1891406

[105] Sarathy JP, Lee E, Dartois V. 2013. Polyamines inhibit porin-mediated fluoroquinolone uptake in mycobacteria. PLoS One 8:e65806. doi:10.1371/journal.pone.0065806 23755283 PMC3670895

[106] Dela Vega AL, Delcour AH. 1996. Polyamines decrease Escherichia coli outer membrane permeability. J Bacteriol 178:3715–3721. doi:10.1128/jb.178.13.3715-3721.1996 8682771 PMC232627

[107] Harmon DE, Ruiz C. 2022. The multidrug efflux regulator AcrR of Escherichia coli responds to exogenous and endogenous ligands to regulate efflux and detoxification. mSphere 7:e0047422. doi:10.1128/msphere.00474-22 36416552 PMC9769551

[108] Chan YY, Chua KL. 2010. Growth-related changes in intracellular spermidine and its effect on efflux pump expression and quorum sensing in Burkholderia pseudomallei. Microbiology (Reading) 156:1144–1154. doi:10.1099/mic.0.032888-0 20035006

[109] Kashiwagi K, Tsuhako MH, Sakata K, Saisho T, Igarashi A, da Costa SO, Igarashi K. 1998. Relationship between spontaneous aminoglycoside resistance in Escherichia coli and a decrease in oligopeptide binding protein. J Bacteriol 180:5484–5488. doi:10.1128/JB.180.20.5484-5488.1998 9765586 PMC107603

[110] Bernier SP, Létoffé S, Delepierre M, Ghigo J-M. 2011. Biogenic ammonia modifies antibiotic resistance at a distance in physically separated bacteria. Mol Microbiol 81:705–716. doi:10.1111/j.1365-2958.2011.07724.x 21651627

[111] Katsu T, Nakagawa H, Yasuda K. 2002. Interaction between polyamines and bacterial outer membranes as investigated with ion-selective electrodes. Antimicrob Agents Chemother 46:1073–1079. doi:10.1128/AAC.46.4.1073-1079.2002 11897592 PMC127117

[112] El-Halfawy OM, Valvano MA. 2013. Chemical communication of antibiotic resistance by a highly resistant subpopulation of bacterial cells. PLoS One 8:e68874. doi:10.1371/journal.pone.0068874 23844246 PMC3700957

[113] Kwon DH, Lu CD. 2006. Polyamines induce resistance to cationic peptide, aminoglycoside, and quinolone antibiotics in Pseudomonas aeruginosa PAO1. Antimicrob Agents Chemother 50:1615–1622. doi:10.1128/AAC.50.5.1615-1622.2006 16641426 PMC1472189

[114] Li J, Beuerman R, Verma CS. 2020. Mechanism of polyamine induced colistin resistance through electrostatic networks on bacterial outer membranes. Biochim Biophys Acta Biomembr 1862:183297. doi:10.1016/j.bbamem.2020.183297 32339485

[115] Bitonti AJ, McCann PP, Sjoerdsma A. 1982. Restriction of bacterial growth by inhibition of polyamine biosynthesis by using monofluoromethylornithine, difluoromethylarginine and dicyclohexylammonium sulphate. Biochem J 208:435–441. doi:10.1042/bj2080435 6818954 PMC1153981

[116] Paulin L, Lindberg LA, Pösö H. 1986. Reversible inhibition of flagella formation after specific inhibition of spermidine synthesis by dicyclohexylamine in Pseudomonas aeruginosa. Antonie Van Leeuwenhoek 52:483–490. doi:10.1007/BF00423409 3101591

[117] Libby PR, Porter CW. 1992. Inhibition of enzymes of polyamine back-conversion by pentamidine and berenil. Biochem Pharmacol 44:830–832. doi:10.1016/0006-2952(92)90424-h 1510731

[118] Lin J, Cheng J, Wang Y, Shen X. 2018. The Pseudomonas Quinolone Signal (PQS): not just for quorum sensing anymore. Front Cell Infect Microbiol 8:230. doi:10.3389/fcimb.2018.00230 30023354 PMC6039570

[119] Rampioni G, Falcone M, Heeb S, Frangipani E, Fletcher MP, Dubern J-F, Visca P, Leoni L, Cámara M, Williams P. 2016. Unravelling the genome-wide contributions of specific 2-Alkyl-4-quinolones and PqsE to quorum sensing in Pseudomonas aeruginosa. PLoS Pathog 12:e1006029. doi:10.1371/journal.ppat.1006029 27851827 PMC5112799

[120] Collier DN, Anderson L, McKnight SL, Noah TL, Knowles M, Boucher R, Schwab U, Gilligan P, Pesci EC. 2002. A bacterial cell to cell signal in the lungs of cystic fibrosis patients. FEMS Microbiol Lett 215:41–46. doi:10.1111/j.1574-6968.2002.tb11367.x 12393198

[121] Abdalla MY, Hoke T, Seravalli J, Switzer BL, Bavitz M, Fliege JD, Murphy PJ, Britigan BE. 2017. Pseudomonas quinolone signal induces oxidative stress and inhibits heme oxygenase-1 expression in lung epithelial cells. Infect Immun 85:e00176-17. doi:10.1128/IAI.00176-17 28630072 PMC5563587

[122] Guina T, Purvine SO, Yi EC, Eng J, Goodlett DR, Aebersold R, Miller SI. 2003. Quantitative proteomic analysis indicates increased synthesis of a quinolone by Pseudomonas aeruginosa isolates from cystic fibrosis airways. Proc Natl Acad Sci USA 100:2771–2776. doi:10.1073/pnas.0435846100 12601166 PMC151416

[123] Aendekerk S, Diggle SP, Song Z, Høiby N, Cornelis P, Williams P, Cámara M. 2005. The MexGHI-OpmD multidrug efflux pump controls growth, antibiotic susceptibility and virulence in Pseudomonas aeruginosa via 4-quinolone-dependent cell-to-cell communication. Microbiology (Reading) 151:1113–1125. doi:10.1099/mic.0.27631-0 15817779

[124] Häussler S, Becker T. 2008. The Pseudomonas Quinolone Signal (PQS) balances life and death in Pseudomonas aeruginosa populations. PLoS Pathog 4:e1000166. doi:10.1371/journal.ppat.1000166 18818733 PMC2533401

[125] Morales-Soto N, Dunham SJB, Baig NF, Ellis JF, Madukoma CS, Bohn PW, Sweedler JV, Shrout JD. 2018. Spatially dependent alkyl quinolone signaling responses to antibiotics in Pseudomonas aeruginosa swarms. J Biol Chem 293:9544–9552. doi:10.1074/jbc.RA118.002605 29588364 PMC6005435

[126] Wei Q, Tarighi S, Dötsch A, Häussler S, Müsken M, Wright VJ, Cámara M, Williams P, Haenen S, Boerjan B, Bogaerts A, Vierstraete E, Verleyen P, Schoofs L, Willaert R, De Groote VN, Michiels J, Vercammen K, Crabbé A, Cornelis P. 2011. Phenotypic and genome-wide analysis of an antibiotic-resistant small colony variant (SCV) of Pseudomonas aeruginosa. PLoS One 6:e29276. doi:10.1371/journal.pone.0029276 22195037 PMC3240657

[127] Sakhtah H, Koyama L, Zhang Y, Morales DK, Fields BL, Price-Whelan A, Hogan DA, Shepard K, Dietrich LEP. 2016. The Pseudomonas aeruginosa efflux pump MexGHI-OpmD transports a natural phenazine that controls gene expression and biofilm development. Proc Natl Acad Sci USA 113:E3538–E3547. doi:10.1073/pnas.1600424113 27274079 PMC4922186

[128] Wolloscheck D, Krishnamoorthy G, Nguyen J, Zgurskaya HI. 2018. Kinetic control of quorum sensing in Pseudomonas aeruginosa by multidrug efflux pumps. ACS Infect Dis 4:185–195. doi:10.1021/acsinfecdis.7b00160 29115136 PMC5807214

[129] Skindersoe ME, Alhede M, Phipps R, Yang L, Jensen PO, Rasmussen TB, Bjarnsholt T, Tolker-Nielsen T, Høiby N, Givskov M. 2008. Effects of antibiotics on quorum sensing in Pseudomonas aeruginosa. Antimicrob Agents Chemother 52:3648–3663. doi:10.1128/AAC.01230-07 18644954 PMC2565867

[130] Florez C, Raab JE, Cooke AC, Schertzer JW. 2017. Membrane distribution of the Pseudomonas quinolone signal modulates outer membrane vesicle production in Pseudomonas aeruginosa. mBio 8:e01034-17. doi:10.1128/mBio.01034-17 28790210 PMC5550756

[131] D’Argenio DA, Calfee MW, Rainey PB, Pesci EC. 2002. Autolysis and autoaggregation in Pseudomonas aeruginosa colony morphology mutants. J Bacteriol 184:6481–6489. doi:10.1128/JB.184.23.6481-6489.2002 12426335 PMC135425

[132] Nguyen D, Joshi-Datar A, Lepine F, Bauerle E, Olakanmi O, Beer K, McKay G, Siehnel R, Schafhauser J, Wang Y, Britigan BE, Singh PK. 2011. Active starvation responses mediate antibiotic tolerance in biofilms and nutrient-limited bacteria. Science 334:982–986. doi:10.1126/science.1211037 22096200 PMC4046891

[133] Pezzoni M, Meichtry M, Pizarro RA, Costa CS. 2015. Role of the Pseudomonas Quinolone Signal (PQS) in sensitising Pseudomonas aeruginosa to UVA radiation. J Photochem Photobiol B: Biol 142:129–140. doi:10.1016/j.jphotobiol.2014.11.014 25535873

[134] Shen L, Ma Y, Liang H. 2012. Characterization of a novel gene related to antibiotic susceptibility in Pseudomonas aeruginosa. J Antibiot 65:59–65. doi:10.1038/ja.2011.111 22146126

[135] Viducic D, Murakami K, Amoh T, Ono T, Miyake Y. 2016. RpoN modulates carbapenem tolerance in Pseudomonas aeruginosa through Pseudomonas quinolone signal and PqsE. Antimicrob Agents Chemother 60:5752–5764. doi:10.1128/AAC.00260-16 27431228 PMC5038263

[136] Viducic D, Murakami K, Amoh T, Ono T, Miyake Y. 2017. Role of the interplay between quorum sensing regulator VqsR and the Pseudomonas quinolone signal in mediating carbapenem tolerance in Pseudomonas aeruginosa. Res Microbiol 168:450–460. doi:10.1016/j.resmic.2017.02.007 28263907

[137] Zhang Y, Wang L, Chen L, Zhu P, Huang N, Chen T, Chen L, Wang Z, Liao W, Cao J, Zhou T. 2022. Novel insight of transcription factor PtrA on pathogenicity and carbapenems resistance in Pseudomonas aeruginosa. Infect Drug Resist 15:4213–4227. doi:10.2147/IDR.S371597 35959145 PMC9359796

[138] Cummins J, Reen FJ, Baysse C, Mooij MJ, O’Gara F. 2009. Subinhibitory concentrations of the cationic antimicrobial peptide colistin induce the pseudomonas quinolone signal in Pseudomonas aeruginosa. Microbiology (Reading) 155:2826–2837. doi:10.1099/mic.0.025643-0 19477905

[139] Yang F, Zhou Y, Bai Y, Pan X, Ha UH, Cheng Z, Wu W, Jin Y, Bai F. 2023. MvfR controls tolerance to polymyxin B by regulating rfaD in Pseudomonas aeruginosa. Microbiol Spectr 11:e0042623. doi:10.1128/spectrum.00426-23 37039709 PMC10269820

[140] Mashburn LM, Whiteley M. 2005. Membrane vesicles traffic signals and facilitate group activities in a prokaryote. Nature 437:422–425. doi:10.1038/nature03925 16163359

[141] Cooke AC, Nello AV, Ernst RK, Schertzer JW. 2019. Analysis of Pseudomonas aeruginosa biofilm membrane vesicles supports multiple mechanisms of biogenesis. PLoS One 14:e0212275. doi:10.1371/journal.pone.0212275 30763382 PMC6375607

[142] Horspool AM, Schertzer JW. 2018. Reciprocal cross-species induction of outer membrane vesicle biogenesis via secreted factors. Sci Rep 8:9873. doi:10.1038/s41598-018-28042-4 29959355 PMC6026191

[143] Schertzer JW, Whiteley M. 2012. A bilayer-couple model of bacterial outer membrane vesicle biogenesis. mBio 3:e00297-11. doi:10.1128/mBio.00297-11 22415005 PMC3312216

[144] Park AJ, Murphy K, Surette MD, Bandoro C, Krieger JR, Taylor P, Khursigara CM. 2015. Tracking the dynamic relationship between cellular systems and extracellular subproteomes in Pseudomonas aeruginosa biofilms. J Proteome Res 14:4524–4537. doi:10.1021/acs.jproteome.5b00262 26378716

[145] Kulkarni HM, Nagaraj R, Jagannadham MV. 2015. Protective role of E. coli outer membrane vesicles against antibiotics. Microbiol Res 181:1–7. doi:10.1016/j.micres.2015.07.008 26640046

[146] Bru J-L, Rawson B, Trinh C, Whiteson K, Høyland-Kroghsbo NM, Siryaporn A. 2019. PQS produced by the Pseudomonas aeruginosa stress response repels swarms away from bacteriophage and antibiotics. J Bacteriol 201:e00383-19. doi:10.1128/JB.00383-19 31451543 PMC6832071

[147] Cao T, Sweedler JV, Bohn PW, Shrout JD. 2020. Spatiotemporal distribution of Pseudomonas aeruginosa alkyl quinolones under metabolic and competitive stress. mSphere 5:e00426-20. doi:10.1128/mSphere.00426-20 32699119 PMC7376503

[148] Déziel E, Lépine F, Milot S, He J, Mindrinos MN, Tompkins RG, Rahme LG. 2004. Analysis of Pseudomonas aeruginosa 4-hydroxy-2-alkylquinolines (HAQs) reveals a role for 4-hydroxy-2-heptylquinoline in cell-to-cell communication. Proc Natl Acad Sci USA 101:1339–1344. doi:10.1073/pnas.0307694100 14739337 PMC337054

[149] Reen FJ, Mooij MJ, Holcombe LJ, McSweeney CM, McGlacken GP, Morrissey JP, O’Gara F. 2011. The Pseudomonas Quinolone Signal (PQS), and its precursor HHQ, modulate interspecies and interkingdom behaviour. FEMS Microbiol Ecol 77:413–428. doi:10.1111/j.1574-6941.2011.01121.x 21539583

[150] Fugère A, Lalonde Séguin D, Mitchell G, Déziel E, Dekimpe V, Cantin AM, Frost E, Malouin F. 2014. Interspecific small molecule interactions between clinical isolates of Pseudomonas aeruginosa and Staphylococcus aureus from adult cystic fibrosis patients. PLoS One 9:e86705. doi:10.1371/journal.pone.0086705 24466207 PMC3900594

[151] Abe-Yoshizumi R, Kamei U, Yamada A, Kimura M, Ichihara S. 2004. The evolution of the phenylacetic acid degradation pathway in bacteria. Biosci Biotechnol Biochem 68:746–748. doi:10.1271/bbb.68.746 15056912

[152] Müller GL, Tuttobene M, Altilio M, Martínez Amezaga M, Nguyen M, Cribb P, Cybulski LE, Ramírez MS, Altabe S, Mussi MA. 2017. Light modulates metabolic pathways and other novel physiological traits in the human pathogen Acinetobacter baumannii. J Bacteriol 199:e00011-17. doi:10.1128/JB.00011-17 28289081 PMC5405214

[153] Ohneck EJ, Arivett BA, Fiester SE, Wood CR, Metz ML, Simeone GM, Actis LA. 2018. Mucin acts as a nutrient source and a signal for the differential expression of genes coding for cellular processes and virulence factors in Acinetobacter baumannii. PLoS One 13:e0190599. doi:10.1371/journal.pone.0190599 29309434 PMC5757984

[154] Alkasir R, Ma Y, Liu F, Li J, Lv N, Xue Y, Hu Y, Zhu B. 2018. Characterization and transcriptome analysis of Acinetobacter baumannii persister cells. Microb Drug Resist 24:1466–1474. doi:10.1089/mdr.2017.0341 29902105

[155] Crofts TS, Wang B, Spivak A, Gianoulis TA, Forsberg KJ, Gibson MK, Johnsky LA, Broomall SM, Rosenzweig CN, Skowronski EW, Gibbons HS, Sommer MOA, Dantas G. 2018. Shared strategies for β-lactam catabolism in the soil microbiome. Nat Chem Biol 14:556–564. doi:10.1038/s41589-018-0052-1 29713061 PMC5964007

[156] Rumbo-Feal S, Gómez MJ, Gayoso C, Álvarez-Fraga L, Cabral MP, Aransay AM, Rodríguez-Ezpeleta N, Fullaondo A, Valle J, Tomás M, Bou G, Poza M. 2013. Whole transcriptome analysis of Acinetobacter baumannii assessed by RNA-sequencing reveals different mRNA expression profiles in biofilm compared to planktonic cells. PLoS One 8:e72968. doi:10.1371/journal.pone.0072968 24023660 PMC3758355

[157] Sugawara S, Mashiguchi K, Tanaka K, Hishiyama S, Sakai T, Hanada K, Kinoshita-Tsujimura K, Yu H, Dai X, Takebayashi Y, Takeda-Kamiya N, Kakimoto T, Kawaide H, Natsume M, Estelle M, Zhao Y, Hayashi K-I, Kamiya Y, Kasahara H. 2015. Distinct characteristics of indole-3-acetic acid and phenylacetic acid, two common auxins in plants. Plant Cell Physiol 56:1641–1654. doi:10.1093/pcp/pcv088 26076971 PMC4523386

[158] Kunkel BN, Harper CP. 2018. The roles of auxin during interactions between bacterial plant pathogens and their hosts. J Exp Bot 69:245–254. doi:10.1093/jxb/erx447 29272462

[159] Krishnamoorthy NK, Kalyan M, Hediyal TA, Anand N, Kendaganna PH, Pendyala G, Yelamanchili SV, Yang J, Chidambaram SB, Sakharkar MK, Mahalakshmi AM. 2024. Role of the gut bacteria-derived metabolite phenylacetylglutamine in health and diseases. ACS Omega 9:3164–3172. doi:10.1021/acsomega.3c08184 38284070 PMC10809373

[160] Bhuiyan MS, Ellett F, Murray GL, Kostoulias X, Cerqueira GM, Schulze KE, Mahamad Maifiah MH, Li J, Creek DJ, Lieschke GJ, Peleg AY. 2016. Acinetobacter baumannii phenylacetic acid metabolism influences infection outcome through a direct effect on neutrophil chemotaxis. Proc Natl Acad Sci USA 113:9599–9604. doi:10.1073/pnas.1523116113 27506797 PMC5003227

[161] Musthafa KS, Sivamaruthi BS, Pandian SK, Ravi AV. 2012. Quorum sensing inhibition in Pseudomonas aeruginosa PAO1 by antagonistic compound phenylacetic acid. Curr Microbiol 65:475–480. doi:10.1007/s00284-012-0181-9 22782469

[162] Pribytkova T, Lightly TJ, Kumar B, Bernier SP, Sorensen JL, Surette MG, Cardona ST. 2014. The attenuated virulence of a Burkholderia cenocepacia paaABCDE mutant is due to inhibition of quorum sensing by release of phenylacetic acid. Mol Microbiol 94:522–536. doi:10.1111/mmi.12771 25155974

[163] Ross BN, Evans E, Whiteley M. 2024. Phenylacetic acid metabolic genes are associated with Mycobacteroides abscessus dominant circulating clone 1. Microbiol Spectr 12:e0133024. doi:10.1128/spectrum.01330-24 39315786 PMC11537035

[164] Kashyap S, Sharma P, Capalash N. 2021. Potential genes associated with survival of Acinetobacter baumannii under ciprofloxacin stress. Microbes Infect 23:104844. doi:10.1016/j.micinf.2021.104844 34098109

[165] Sass A, Marchbank A, Tullis E, Lipuma JJ, Mahenthiralingam E. 2011. Spontaneous and evolutionary changes in the antibiotic resistance of Burkholderia cenocepacia observed by global gene expression analysis. BMC Genomics 12:373. doi:10.1186/1471-2164-12-373 21781329 PMC3155924

[166] Mazumdar K, Dutta NK, Dastidar SG, Motohashi N, Shirataki Y. 2006. Diclofenac in the management of E. coli urinary tract infections. In Vivo 20:613–619. 17091768

[167] Bisaro F, Jackson-Litteken CD, McGuffey JC, Hooppaw AJ, Bodrog S, Jebeli L, Janet-Maitre M, Ortiz-Marquez JC, van Opijnen T, Scott NE, Di Venanzio G, Feldman MF. 2024. Diclofenac sensitizes multi-drug resistant Acinetobacter baumannii to colistin. PLoS Pathog 20:e1012705. doi:10.1371/journal.ppat.1012705 39571043 PMC11620633

[168] Lee J-H, Lee J. 2010. Indole as an intercellular signal in microbial communities. FEMS Microbiol Rev 34:426–444. doi:10.1111/j.1574-6976.2009.00204.x 20070374

[169] Hu Y, Kwan BW, Osbourne DO, Benedik MJ, Wood TK. 2015. Toxin YafQ increases persister cell formation by reducing indole signalling. Environ Microbiol 17:1275–1285. doi:10.1111/1462-2920.12567 25041421

[170] Kawamura-Sato K, Shibayama K, Horii T, Iimuma Y, Arakawa Y, Ohta M. 1999. Role of multiple efflux pumps in Escherichia coli in indole expulsion. FEMS Microbiol Lett 179:345–352. doi:10.1111/j.1574-6968.1999.tb08748.x 10518736

[171] Gaimster H, Cama J, Hernández-Ainsa S, Keyser UF, Summers DK. 2014. The indole pulse: a new perspective on indole signalling in Escherichia coli. PLoS One 9:e93168. doi:10.1371/journal.pone.0093168 24695245 PMC3973702

[172] Zarkan A, Liu J, Matuszewska M, Gaimster H, Summers DK. 2020. Local and universal action: the paradoxes of indole signalling in bacteria. Trends Microbiol 28:566–577. doi:10.1016/j.tim.2020.02.007 32544443

[173] Darkoh C, Chappell C, Gonzales C, Okhuysen P. 2015. A rapid and specific method for the detection of indole in complex biological samples. Appl Environ Microbiol 81:8093–8097. doi:10.1128/AEM.02787-15 26386049 PMC4651089

[174] Anderson GM. 2021. “The quantitative determination of indolic microbial tryptophan metabolites in human and rodent samples: a systematic review”. J Chromatogr B 1186:123008. doi:10.1016/j.jchromb.2021.123008 34735972

[175] Zarkan A, Matuszewska M, Trigg SB, Zhang M, Belgami D, Croft C, Liu J, El-Ouisi S, Greenhalgh J, Duboff JS, Rahman T, Summers DK. 2020. Inhibition of indole production increases the activity of quinolone antibiotics against E. coli persisters. Sci Rep 10:11742. doi:10.1038/s41598-020-68693-w 32678197 PMC7366635

[176] Field CM, Summers DK. 2012. Indole inhibition of ColE1 replication contributes to stable plasmid maintenance. Plasmid 67:88–94. doi:10.1016/j.plasmid.2011.11.004 22172706

[177] Lang M, Krin E, Korlowski C, Sismeiro O, Varet H, Coppée J-Y, Mazel D, Baharoglu Z. 2021. Sleeping ribosomes: bacterial signaling triggers RaiA mediated persistence to aminoglycosides. iScience 24:103128. doi:10.1016/j.isci.2021.103128 34611612 PMC8476650

[178] Goode O, Smith A, Zarkan A, Cama J, Invergo BM, Belgami D, Caño-Muñiz S, Metz J, O’Neill P, Jeffries A, Norville IH, David J, Summers D, Pagliara S. 2021. Persister Escherichia coli cells have a lower intracellular ph than susceptible cells but maintain their pH in response to antibiotic treatment. mBio 12:e0090921. doi:10.1128/mBio.00909-21 34281389 PMC8406257

[179] Zarkan A, Caño-Muñiz S, Zhu J, Al Nahas K, Cama J, Keyser UF, Summers DK. 2019. Indole pulse signalling regulates the cytoplasmic pH of E. coli in a memory-like manner. Sci Rep 9:3868. doi:10.1038/s41598-019-40560-3 30846797 PMC6405993

[180] Song S, Wood TK. 2020. Combatting persister cells with substituted indoles. Front Microbiol 11:1565. doi:10.3389/fmicb.2020.01565 32733426 PMC7358577

[181] Lee HH, Molla MN, Cantor CR, Collins JJ. 2010. Bacterial charity work leads to population-wide resistance. Nature 467:82–85. doi:10.1038/nature09354 20811456 PMC2936489

[182] Lee J, Zhang XS, Hegde M, Bentley WE, Jayaraman A, Wood TK. 2008. Indole cell signaling occurs primarily at low temperatures in Escherichia coli. ISME J 2:1007–1023. doi:10.1038/ismej.2008.54 18528414

[183] Hirakawa H, Inazumi Y, Masaki T, Hirata T, Yamaguchi A. 2005. Indole induces the expression of multidrug exporter genes in Escherichia coli. Mol Microbiol 55:1113–1126. doi:10.1111/j.1365-2958.2004.04449.x 15686558

[184] Yaikhan T, Chuerboon M, Tippayatham N, Atimuttikul N, Nuidate T, Yingkajorn M, Tun AW, Buncherd H, Tansila N. 2019. Indole and derivatives modulate biofilm formation and antibiotic tolerance of Klebsiella pneumoniae. Indian J Microbiol 59:460–467. doi:10.1007/s12088-019-00830-0 31762509 PMC6842365

[185] Lee J, Attila C, Cirillo SLG, Cirillo JD, Wood TK. 2009. Indole and 7-hydroxyindole diminish Pseudomonas aeruginosa virulence. Microb Biotechnol 2:75–90. doi:10.1111/j.1751-7915.2008.00061.x 21261883 PMC3815423

[186] Salama GG, El-Mahdy TS, Moustafa WH, Emara M. 2024. Downregulation of Klebsiella pneumoniae RND efflux pump genes following indole signal produced by Escherichia coli. BMC Microbiol 24:312. doi:10.1186/s12866-024-03443-w 39182027 PMC11344464

[187] Wu T, Wilhelm MJ, Li Y, Ma J, Dai HL. 2022. Indole facilitates antimicrobial uptake in bacteria. ACS Infect Dis 8:1124–1133. doi:10.1021/acsinfecdis.1c00618 35297612

[188] Brauner A, Fridman O, Gefen O, Balaban NQ. 2016. Distinguishing between resistance, tolerance and persistence to antibiotic treatment. Nat Rev Microbiol 14:320–330. doi:10.1038/nrmicro.2016.34 27080241

[189] Mehboob J, Herman R, Elston RC, Afolabi H, Kinniment-Williams BE, van der Woude MW, Wilkinson AJ, Thomas GH. 2025. Itaconate utilisation by the human pathogen Pseudomonas aeruginosa requires uptake via the IctPQM TRAP transporter. Biochem J 482:1277–1288. doi:10.1042/BCJ20253132 40891131 PMC12599236

[190] Riquelme SA, Liimatta K, Wong Fok Lung T, Fields B, Ahn D, Chen D, Lozano C, Sáenz Y, Uhlemann A-C, Kahl BC, Britto CJ, DiMango E, Prince A. 2020. Pseudomonas aeruginosa utilizes host-derived itaconate to redirect its metabolism to promote biofilm formation. Cell Metab 31:1091–1106. doi:10.1016/j.cmet.2020.04.017 32428444 PMC7272298

[191] Singh S, Singh PK, Jha A, Naik P, Joseph J, Giri S, Kumar A. 2021. Integrative metabolomics and transcriptomics identifies itaconate as an adjunct therapy to treat ocular bacterial infection. Cell Rep Med 2:100277. doi:10.1016/j.xcrm.2021.100277 34095879 PMC8149370

[192] Moulding PB, El-Halfawy OM. 2024. Chemical-mediated virulence: the effects of host chemicals on microbial virulence and potential new antivirulence strategies. Can J Microbiol 70:405–425. doi:10.1139/cjm-2024-0017 38905704

[193] Crabbé A, Ostyn L, Staelens S, Rigauts C, Risseeuw M, Dhaenens M, Daled S, Van Acker H, Deforce D, Van Calenbergh S, Coenye T. 2019. Host metabolites stimulate the bacterial proton motive force to enhance the activity of aminoglycoside antibiotics. PLoS Pathog 15:e1007697. doi:10.1371/journal.ppat.1007697 31034512 PMC6508747

[194] Luan HH, Medzhitov R. 2016. Food fight: role of itaconate and other metabolites in antimicrobial defense. Cell Metab 24:379–387. doi:10.1016/j.cmet.2016.08.013 27626199 PMC5024735

[195] Al Akiki Dit Al Mazraani R, Malys N, Maliene V. 2025. Itaconate and its derivatives as anti-pathogenic agents. RSC Adv 15:4408–4420. doi:10.1039/d4ra08298b 39931396 PMC11808480

[196] Huang Q, Duan C, Ma H, Nong C, Zheng Q, Zhou J, Zhao N, Mou X, Liu T, Zou S, Yang N, Tong A, Qin W, Bao R. 2024. Structural and functional characterization of itaconyl-CoA hydratase and citramalyl-CoA lyase involved in itaconate metabolism of Pseudomonas aeruginosa. Structure 32:941–952. doi:10.1016/j.str.2024.04.004 38677288

[197] Zhao R, Xu L, Chen J, Yang Y, Guo X, Dai M, Tian G-B, Qin L-N. 2024. Itaconate induces tolerance of Staphylococcus aureus to aminoglycoside antibiotics. Front Microbiol 15:2024. doi:10.3389/fmicb.2024.1450085 PMC11471559 39403084

[198] Mora L de O, Antunes LMG, Francescato HDC, Bianchi M de LP. 2003. The effects of oral glutamine on cisplatin-induced nephrotoxicity in rats. Pharmacol Res 47:517–522. doi:10.1016/s1043-6618(03)00040-9 12742005

[199] Aoki M, Mochizuki M, Okamura T, Hatayama K, Nakamura A, Morishita K. 2014. A 4-week oral toxicity study of L-alanine in rats with a recovery period of 2 weeks. Fundam Toxicol Sci 1:63–72. doi:10.2131/fts.1.63

[200] Schulz-Bohm K, Martín-Sánchez L, Garbeva P. 2017. Microbial volatiles: small molecules with an important role in intra- and inter-kingdom interactions. Front Microbiol 8:2484. doi:10.3389/fmicb.2017.02484 29312193 PMC5733050

[201] Kemmler E, Lemfack MC, Goede A, Gallo K, Toguem SMT, Ahmed W, Millberg I, Preissner S, Piechulla B, Preissner R. 2025. mVOC 4.0: a database of microbial volatiles. Nucleic Acids Res 53:D1692–D1696. doi:10.1093/nar/gkae961 39475188 PMC11701663

[202] Lemfack MC, Gohlke B-O, Toguem SMT, Preissner S, Piechulla B, Preissner R. 2018. mVOC 2.0: a database of microbial volatiles. Nucleic Acids Res 46:D1261–D1265. doi:10.1093/nar/gkx1016 29106611 PMC5753297

[203] Shatalin K, Shatalina E, Mironov A, Nudler E. 2011. H_2_S: a universal defense against antibiotics in bacteria. Science 334:986–990. doi:10.1126/science.1209855 22096201

[204] Caruso L, Mellini M, Catalano Gonzaga O, Astegno A, Forte E, Di Matteo A, Giuffrè A, Visca P, Imperi F, Leoni L, Rampioni G. 2024. Hydrogen sulfide production does not affect antibiotic resistance in Pseudomonas aeruginosa. Antimicrob Agents Chemother 68:e0007524. doi:10.1128/aac.00075-24 38445869 PMC10989007

[205] Weikum J, Ritzmann N, Jelden N, Klöckner A, Herkersdorf S, Josten M, Sahl H-G, Grein F. 2018. Sulfide protects Staphylococcus aureus from aminoglycoside antibiotics but cannot be regarded as a general defense mechanism against antibiotics. Antimicrob Agents Chemother 62:e00602-18. doi:10.1128/AAC.00602-18 30061290 PMC6153802

[206] Xuan G, Lü C, Xu H, Chen Z, Li K, Liu H, Liu H, Xia Y, Xun L. 2020. Sulfane sulfur is an intrinsic signal activating MexR-regulated antibiotic resistance in Pseudomonas aeruginosa. Mol Microbiol 114:1038–1048. doi:10.1111/mmi.14593 32875640

[207] Ono K, Kitamura Y, Zhang T, Tsutsuki H, Rahman A, Ihara T, Akaike T, Sawa T. 2021. Cysteine hydropersulfide inactivates β-lactam antibiotics with formation of ring-opened carbothioic S-acids in bacteria. ACS Chem Biol 16:731–739. doi:10.1021/acschembio.1c00027 33781062

[208] Winterbourn CC, Kettle AJ. 2013. Redox reactions and microbial killing in the neutrophil phagosome. Antioxid Redox Signal 18:642–660. doi:10.1089/ars.2012.4827 22881869

[209] Crane BR, Sudhamsu J, Patel BA. 2010. Bacterial nitric oxide synthases. Annu Rev Biochem 79:445–470. doi:10.1146/annurev-biochem-062608-103436 20370423

[210] Canthaboo C, Xing D, Wei XQ, Corbel MJ. 2002. Investigation of role of nitric oxide in protection from Bordetella pertussis respiratory challenge. Infect Immun 70:679–684. doi:10.1128/IAI.70.2.679-684.2002 11796599 PMC127720

[211] Pacelli R, Wink DA, Cook JA, Krishna MC, DeGraff W, Friedman N, Tsokos M, Samuni A, Mitchell JB. 1995. Nitric oxide potentiates hydrogen peroxide-induced killing of Escherichia coli. J Exp Med 182:1469–1479. doi:10.1084/jem.182.5.1469 7595217 PMC2192188

[212] Marcinkiewicz J. 1997. Nitric oxide and antimicrobial activity of reactive oxygen intermediates. Immunopharmacology 37:35–41. doi:10.1016/s0162-3109(96)00168-3 9285242

[213] Yadav R, Samuni Y, Abramson A, Zeltser R, Casap N, Kabiraj TK, L Banach M, Samuni U. 2014. Pro-oxidative synergic bactericidal effect of NO: kinetics and inhibition by nitroxides. Free Radic Biol Med 67:248–254. doi:10.1016/j.freeradbiomed.2013.10.012 24140438

[214] Gusarov I, Nudler E. 2005. NO-mediated cytoprotection: instant adaptation to oxidative stress in bacteria. Proc Natl Acad Sci USA 102:13855–13860. doi:10.1073/pnas.0504307102 16172391 PMC1236549

[215] Shatalin K, Gusarov I, Avetissova E, Shatalina Y, McQuade LE, Lippard SJ, Nudler E. 2008. Bacillus anthracis-derived nitric oxide is essential for pathogen virulence and survival in macrophages. Proc Natl Acad Sci USA 105:1009–1013. doi:10.1073/pnas.0710950105 18215992 PMC2242674

[216] Hutfless EH, Chaudhari SS, Thomas VC. 2018. Emerging roles of nitric oxide synthase in bacterial physiology. Adv Microb Physiol 72:147–191. doi:10.1016/bs.ampbs.2018.01.006 29778214

[217] Gusarov I, Shatalin K, Starodubtseva M, Nudler E. 2009. Endogenous nitric oxide protects bacteria against a wide spectrum of antibiotics. Science 325:1380–1384. doi:10.1126/science.1175439 19745150 PMC2929644

[218] Létoffé S, Audrain B, Bernier SP, Delepierre M, Ghigo J-M. 2014. Aerial exposure to the bacterial volatile compound trimethylamine modifies antibiotic resistance of physically separated bacteria by raising culture medium pH. mBio 5:e00944-13. doi:10.1128/mBio.00944-13 24399857 PMC3884056

[219] Hettinga KA, van Valenberg HJF, Lam T, van Hooijdonk ACM. 2008. Detection of mastitis pathogens by analysis of volatile bacterial metabolites. J Dairy Sci 91:3834–3839. doi:10.3168/jds.2007-0941 18832205

[220] Rees CA, Smolinska A, Hill JE. 2016. The volatile metabolome of Klebsiella pneumoniae in human blood. J Breath Res 10:027101. doi:10.1088/1752-7155/10/2/027101 27163334

[221] Kim K, Lee S, Ryu C-M. 2013. Interspecific bacterial sensing through airborne signals modulates locomotion and drug resistance. Nat Commun 4:1809. doi:10.1038/ncomms2789 23651997

